# EBV and Apoptosis: The Viral Master Regulator of Cell Fate?

**DOI:** 10.3390/v9110339

**Published:** 2017-11-13

**Authors:** Leah Fitzsimmons, Gemma L. Kelly

**Affiliations:** 1 Institute of Cancer and Genomic Sciences and Centre for Human Virology, College of Medical and Dental Sciences, University of Birmingham, Edgbaston, Birmingham B15 2TT, UK; l.fitzsimmons@bham.ac.uk; 2 Molecular Genetics of Cancer Division, The Walter and Eliza Hall Institute for Medical Research, Parkville, Melbourne, VIC 3052, Australia; 3 Department of Medical Biology, The University of Melbourne, Parkville, Melbourne, VIC 3052, Australia

**Keywords:** EBV, apoptosis, genetic cooperation, latency, virus cancers, p53, BCL-2 family, growth transformation

## Abstract

Epstein–Barr virus (EBV) was first discovered in cells from a patient with Burkitt lymphoma (BL), and is now known to be a contributory factor in 1–2% of all cancers, for which there are as yet, no EBV-targeted therapies available. Like other herpesviruses, EBV adopts a persistent latent infection in vivo and only rarely reactivates into replicative lytic cycle. Although latency is associated with restricted patterns of gene expression, genes are never expressed in isolation; always in groups. Here, we discuss (1) the ways in which the latent genes of EBV are known to modulate cell death, (2) how these mechanisms relate to growth transformation and lymphomagenesis, and (3) how EBV genes cooperate to coordinately regulate key cell death pathways in BL and lymphoblastoid cell lines (LCLs). Since manipulation of the cell death machinery is critical in EBV pathogenesis, understanding the mechanisms that underpin EBV regulation of apoptosis therefore provides opportunities for novel therapeutic interventions.

## 1. Introduction

All viruses possess methods to negotiate and subvert the cell death pathways of their hosts, but for viruses such as influenza that lead to acute infections, the battle for host cell survival is ultimately lost when the host clears the virus. Persistent viruses however, such as those of the herpesvirus family that are carried by the host for life, must avoid elimination by immune cells, whilst continuing to disseminate within the host and avoid being lost through normal cell turnover. Human herpesvirus 4, also known as Epstein–Barr virus (EBV), is extremely efficient at establishing a persistent life-long infection in human B cells. Most primary infections occur asymptomatically in early childhood and by adulthood the majority (95% world-wide) of the adult population are infected with EBV [1]. Somewhat paradoxically, given the ubiquitous and asymptomatic nature of infection, EBV is the archetypal human tumour virus. EBV was first associated with an unusual and aggressive form of childhood lymphoma in 1964 [2,3], and is now known to contribute to over 200,000 new cancer diagnoses each year [4]. These unusual characteristics are, in part, due to the many and various ways EBV has evolved to exhibit exquisite control over cell death. Here, we review the mechanisms by which EBV genes co-operate to regulate cell death and how this may contribute to lymphomagenesis by focussing on evidence from the study of in vitro transformation of B cells and Burkitt lymphoma, the cancer in which EBV was discovered.

The existence of EBV was first suspected by Denis Parsons Burkitt, a surgeon working in post-war Uganda. In 1958, he published the first detailed clinical study of a strange lymphoma affecting the jaws and abdomens of children across sub-Saharan Africa [5]. He was struck by both the prevalence and the poor prognosis of the disease: the tumours accounted for more cases of cancer in childhood than all other malignancies combined and furthermore, were often rapidly fatal [6]. Burkitt lymphoma (BL), as it came to be known, also had an unusual geographical distribution which overlapped almost perfectly with that of arthropod borne infectious diseases such as Yellow Fever and Rift Valley Fever [7,8,9,10]. A chance meeting with the then animal virologist, Anthony Epstein, led to the search for a possible viral cause of BL and in 1964 the first micrographs showing the unmistakable icosahedral structures of a previously undescribed herpesvirus were published [2]. It did however take several more years until Epstein–Barr virus (named after Epstein and his PhD student, Yvonne Barr) was convincingly shown to be an aetiological agent in BL and other cancers (reviewed in detail elsewhere [11,12]).

## 2. ‘Transforming’ Cell Death: Latent Genes

**Figure 1:**
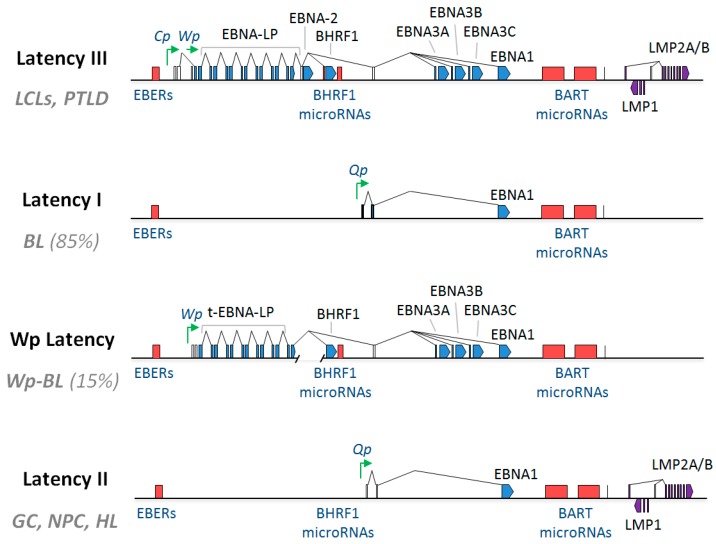
Patterns of latent gene expression found in Epstein–Barr virus (EBV)-associated malignancies and growth transformed B cell lines. Schematic showing: the Latency III EBV gene expression programme, as found in B cells transformed in vitro into lymphoblastoid cell lines (LCLs); Latency I EBV gene expression as found in the majority (85%) of EBV-positive Burkitt lymphomas (BL); Wp-restricted latency (Wp Latency), as found in a minority (15%) of EBV-positive BLs (termed Wp-BL); and Latency II EBV gene expression, which is found in EBV-positive Hodgkin lymphoma (HL) as well as the EBV-associated epithelial malignancies, nasopharyngeal carcinoma (NPC) and gastric carcinoma (GC). Latent proteins (EBNA1, EBNA2, EBNA3A, EBNA3B, EBNA3C, EBNA-LP, BHRF1, LMP1 and LMP2A/B) are shown in blue. Non-coding RNAs (EBERs, miR-BHRF1s and miR-BARTs) are shown in red, and selected latent promoters (Cp, Wp and Qp) are shown in green. Connecting lines denote splicing patterns, whilst blocks indicate exons. In Wp-BL, EBNA-LP is truncated due to a genomic deletion and is therefore denoted as t-EBNA-LP.

The oncogenic potential of EBV was first demonstrated experimentally in 1967, when it was observed that co-culturing lethally-irradiated, EBV-producing BL cells with primary human lymphocytes led to the outgrowth of permanently proliferating lymphoblastoid cell lines (LCLs) [13,14]. This process of growth transformation, by which EBV is able to immortalise B lymphocytes that would otherwise senesce and die in vitro, provided an important model for the study of EBV infection and has therefore been extensively researched. Clearly, the activation of resting B cells into cell cycle is imperative for growth transformation, but inhibition of cell death is equally essential. Although the virus encodes around 100 open reading frames (ORFs), the vast majority of EBV genes have replicative, immune suppressive or structural functions and as such are only expressed during the viral lytic cycle [1]. EBV establishes a largely latent infection in infected cells (the virus is lytic in around 1% of cells at any given time), where it is maintained as an episome by virtue of its ability to ‘piggy back’ onto the host replication machinery during mitosis [15,16]. During these latent infections, only a small subset of EBV-encoded gene products is expressed [17,18]. In established LCLs (immortalised B cells) EBV displays a Latency III pattern of infection. Latency III is the most extensive form of latent infection, involving the expression of ten EBV-encoded proteins and a variety of non-coding RNAs. These are the Epstein–Barr Nuclear Antigens (EBNA-1, EBNA-2, EBNAs-3A, -3B, -3C and EBNA-LP), Latent Membrane Proteins (LMP1, LMP2A and LMP2B), and the viral BCL-2 homologue, BHRF1; as well as two non-coding RNAs (EBER1 and EBER2), and two families of microRNAs encoded within the *BamHI* A rightward transcripts (BARTs) and the BHRF1 locus (BHRF1 miRNAs), respectively (Figure 1) [18,19,20,21,22,23,24]. These EBV latent gene products are expressed at different time points post-infection of B cells, finally leading to growth transformation.

### 2.1. Dynamics of Early Infection

**Figure 2:**
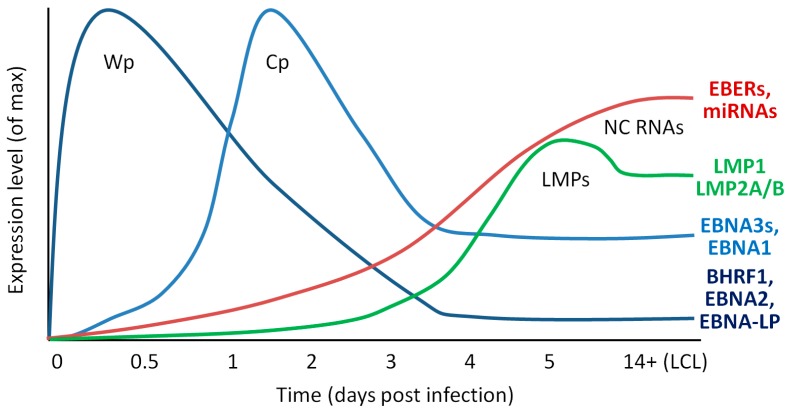
Temporal patterns of latent gene expression during growth transformation of primary resting B cells. Schematic showing the general transcription patterns of different classes of latent EBV genes during in vitro growth transformation of primary, resting B cells. Wp-derived transcripts preferentially give rise to BHRF1, EBNA2 and EBNA-LP in order to kick start cells into cycle, though they also encode EBNA-3A, -3B and -3C (EBNA3s) and EBNA1. Cp can encode all EBNAs and BHRF1. NC RNAs include EBER1, EBER2, miR-BARTs and miR-BHRF1s. Data are cumulative estimations based on transcriptional data published by Tierney et al. [33], Shannon-Lowe et al. [30], and Amoroso et al. [24].

Upon infection of resting B cells, EBV gene expression, driven by host cell RNA polymerase II, begins almost immediately; the Wp promoter that drives early latent gene expression reaches maximal activity around 8–12 h post-infection (PI). These long and differentially spliced Wp-transcripts preferentially encode EBNA-LP, EBNA-2 and BHRF1 [25,26]. The nuclear antigens (EBNAs-LP and -2) then transactivate the Cp and LMP promoters [27,28,29], leading to the expression of EBNA1, EBNA3A, -3B and -3C and LMP1, 2A and 2B, respectively, which reach peak expression at 2–3 days PI [25,30]. Importantly however, there is a delay between maximal expression of latent transcripts and the proteins they encode. The EBNA2, EBNA-LP and BHRF1 proteins reach levels comparable to those in established LCLs at around 72 h [25,31], whereas LMP1 protein is low or undetectable until 5 days PI. [19,32]. Expression of EBV non-coding RNAs is similarly delayed: they are not detected at appreciable levels until several days after infection (Figure 2). Many of these EBV genes are reported to have roles in cell proliferation and/or survival.

### 2.2. EBNA-2 and EBNA-LP

EBNA-2 and EBNA-LP are the first proteins to be expressed following infection of B cells. EBNA2 is a functional mimic of cellular Notch [34,35,36] and is responsible for kick-starting cell cycle activation through its RBP-Jκ-mediated pleiotropic effects on chromatin organisation and gene regulation [37,38,39,40]. Therefore, it is not surprising that EBNA2 expression is essential for B cell transformation [41]. EBNA2 can also inhibit intrinsic cell death through interactions with, and upregulation of, cellular proteins. EBNA-2 can directly bind and inhibit the orphan nuclear receptor Nur77 [42,43] which is reported to bind and modulate the function of several pro-survival BCL-2 family members [44]. Additionally, EBNA-2 expression was shown to upregulate the pro-survival BCL-2 family protein, BFL-1/A1, at the mRNA level via binding to RBP-Jκ/CBF1 [45] and co-ordinately downregulate the BCL-2 family death inducer, BIK [46]. More recently, EBNA-2 has also been shown to contribute to the activation of *MYC*, that can both increase proliferation and sensitise cells to apoptosis, through long-range interactions [47]. EBNA-LP, the transcriptional coactivator of EBNA2 [48], is essential for efficient B cell transformation [49,50,51], but has so far had few survival functions attributed to it in the context of LCLs. Interestingly, however, it has been reported that EBNA-LP (also called EBNA-5), can bind Fte-1/S3a, which is able to contribute to cell survival by interacting with PARP [52]. Another study found that EBNA-LP could interact with p14^ARF^ in a yeast 2-hybrid system and colocalised with p14^ARF^ and p53 transcripts in LCLs [53]. EBNA-LP has also been shown to interact with BCL-2 in the presence of HAX-1 in pull down experiments using glutathione S-transferase fusion proteins in the primate kidney cell line, COS-7 [54]. Therefore, the possible survival functions of EBNA-LP during transformation warrant further investigation.

### 2.3. EBNA-3A, -3B and -3C

The EBNA-3s are a family of three large proteins (3A, 3B, 3C), which likely arose by gene duplication, that predominantly function as regulators of host cell and virus transcription. Like EBNA-2, the EBNA-3 proteins do not bind DNA directly, but instead transactivate or repress gene expression via interactions with transcription factors, such as RBP-Jκ, for which all four EBNAs compete (reviewed in [55]). Although they share less than 30% amino acid identity, EBNA-3A, -3B and -3C exhibit structural similarity [56,57] and display some overlap in terms of some of the loci and processes that they regulate. Interestingly, only EBNA-3C is essential for B cell transformation; although B cells infected with viruses lacking EBNA-3A display a growth impairment and readily undergo apoptosis [58,59,60]. In contrast, EBNA-3B is dispensable for B cell transformation and LCLs generated with an EBNA-3B knock-out (KO) virus exhibit comparable resistance to apoptosis as those transformed with wild-type (wt) EBV [61,62]. Extensive analyses of cells infected with EBNA-3 KO or conditional viruses that encode estrogen-inducible EBNA-3 proteins revealed that EBNA-3A and -3C are able to co-operatively downregulate the apoptosis inducing, BH3-only protein BIM [63,64,65] as well as the tumour suppressors p16^INK4a^ and p14^ARF^ [58,59,66,67,68,69] through epigenetic silencing. EBNA-3C can also reportedly interact with p53 as well as binding and stabilising the p53 regulators, ING4, ING5, MDM2 and Gemin3 [70,71,72]. Although the downregulation of BIM and p14^ARF^ by EBNA-3A and -3C occurs through epigenetic silencing at their transcriptional start sites (TSS) [59,63,64,66,68], the EBNA3 proteins regulate many other genes by long range interactions up to 50 kb away from a TSS [69,73]. It is estimated that collectively, the EBNA-3 proteins can bind more than 7000 sites on the cellular genome; therefore, it is likely that other cell survival genes regulated by the EBNA3s will be identified in the future.

### 2.4. EBNA-1

The primary function of EBNA-1 protein is to tether EBV episomes onto host cell chromatin to ensure the viral DNA is replicated during cell mitosis. Therefore, EBNA1 expression is essential for the maintenance of latent infection [74,75,76]. There is evidence that EBNA-1 may also affect the survival of EBV-infected cells, but interestingly the literature in this regard is conflicting, with reports that EBNA1 is both pro- and anti-apoptotic in function [77]. EBNA-1 has been shown to cause genomic instability by triggering reactive oxygen species production and the DNA damage response (DDR) [78,79]. Conversely however, EBNA1 may stabilise p53 to counteract DDR by binding to the p53 regulator, USP7 [80] and further block downstream caspase activation by upregulation of Survivin [81]. EBNA-1 reportedly binds and regulates the promoters of many other cellular genes but the functional consequences and implications of these interactions for cell survival are not yet fully elucidated [82,83,84,85].

### 2.5. LMP1

LMP1 promotes cell growth and survival and is essential for B cell transformation [86,87]. Functionally, it is a CD40 homologue [88,89] that constitutively mimics cellular TNF signalling through its two cytoplasmic signalling domains, CTAR1 and CTAR2 (sometimes referred to as TES1 and 2) [90,91,92]. CTAR1 binds TRAF and consequently stimulates the non-canonical NF-κB cascade via p100/RelB [93,94], whilst CTAR2 activates both the canonical pathway and JNK signalling through binding to TRADD, IRF7 and BS69 and downstream activation of IKKβ [95,96,97]. Consequently, LMP1 upregulates NF-κB responsive BCL-2 pro-survival proteins, including BCL-2, MCL-1 and BFL-1/A1 [98,99,100,101,102] and the cyto-protective, JNK-regulated chemokines, CCL3 and CCL4 [103]. Additionally, a recent genome-wide CRISPR/Cas9 loss-of-function screen in BLs and LCLs in vitro identified another previously identified NF-κB/LMP1 transcriptional target, cFLIP [104], which can suppress the extrinsic apoptotic and necroptosis pathways, as critically important for the survival of LCLs [105]. Conversely, LMP1 has also been shown to be capable of inducing Fas-mediated apoptosis in B cells with coincident cleavage of Caspase 8 and BID [106,107]. Interestingly, this pro-death activity, which is independent of the CTARs and instead maps to the transmembrane region is masked when LMP1 is expressed at moderate levels and is only evident where LMP1 is over-expressed [98].

### 2.6. LMP2A and LMP2B

LMP2A and 2B are expressed from a series of complex, overlapping transcripts, each driven by its own unique promoter that, like the LMP1p, are transactivated by EBNA-2 during transformation [108,109,110]. LMP2A, like LMP1, is a constitutive mimic of cellular receptor signalling. Whereas LMP1 is a CD40 homologue, LMP2A emulates B cell receptor (BCR) cross-linking, therefore during infection of B cells, the LMPs are able to recapitulate the signals essential for normal B cell development and function [1]. In vitro, LMP2A is dispensable for the initial transformation of B cells [111,112,113,114], but has been shown to provide critical ongoing cell survival signals, including inhibition of TGF-β-associated apoptosis through activation of PI3K/Akt/mTOR [105,115,116]. Furthermore, LMP2A can rescue “crippled” B cells; that is germinal centre B cells with Ig mutations that consequently lack a functional BCR, and so would otherwise rapidly undergo programmed cell death [117,118]. LMP2A function is dependent on recruitment of and interaction with Src family tyrosine kinases and LMP2A must be phosphorylated at residues Y74 and Y85 by Syk in order to activate pro-survival PI3K/Akt/mTOR signalling [119,120,121]. LMP2B shares most of its exons with LMP2A, but lacks the N-terminal Syk domain [110,122]. Much less is known about the function of LMP2B compared to LMP2A, but it has been shown to functionally oppose LMP2A and instead may potentiate LMP1 signalling in order to fine tune signalling in infected B cells [123].

### 2.7. BHRF1 and BALF1

A number of viruses, including EBV, encode homologues of cellular pro-survival BCL-2 family proteins in order to evade apoptosis triggered as part of the anti-viral response. These viral BCL-2 proteins (vBCL-2s) vary in the degree of similarity they display to their cellular counterparts in terms of sequence, structure and function (reviewed in [124]). Although most vBCL-2 proteins are regarded as regulators of the intrinsic apoptosis pathway, some also regulate additional cellular processes [125,126,127,128].

EBV encodes two BCL-2 homologues, called BHRF1 and BALF1. These viral proteins have long been known to be expressed at high levels during lytic cycle, presumably to keep host cells alive to ensure efficient virus replication. They have more recently been shown to also be expressed during the early stages of primary B cell infection in vitro: BHRF1 and BALF1 cDNAs can be detected by day 1 PI [33,129]. Recombinant viruses lacking either BHRF1 or BALF1 are able to transform resting B cells in vitro, but are slightly impaired. However, the loss of both BHRF1 and BALF1 completely abrogates the ability of the virus to transform B cells, suggesting redundancy in their function in this regard [129]. Whilst BHRF1 protein has been shown to also be constitutively expressed in established latent LCLs [19], no antibody to BALF1 exists and therefore the expression of BALF1 protein in LCLs has not been examined. Therefore, although BALF1 transcripts can be detected at low levels in LCLs [33], it is not known whether these arise from a small number of lytically infected cells or, like BHRF1, are expressed during latency.

Both BHRF1 and BALF1 contain all four of the functionally important BCL-2 homology domains, BH1-4 [130,131]. BHRF1 is relatively well-characterised: its BH3 binding groove exhibits 25% amino acid sequence similarity to cellular BCL-2 [132] and structurally most closely resembles BCL-x_L_. Like cellular BCL-2 pro-survival proteins, BHRF1 protein has a hydrophobic groove formed by α-helices 2–5 of its 7-helix bundle [133], into which BH3 ligands can bind. BHRF1 is potently anti-apoptotic in vitro and can confer protection to different cell types from a wide variety of death-inducing stimuli [19,131,133,134,135,136,137,138,139,140,141,142]. Furthermore, ectopic expression of BHRF1 from a retrovirus vector in *Eμ-Myc* mouse lymphoma cells in vivo can confer protection from DNA damaging chemotherapeutics [133].

**Figure 3:**
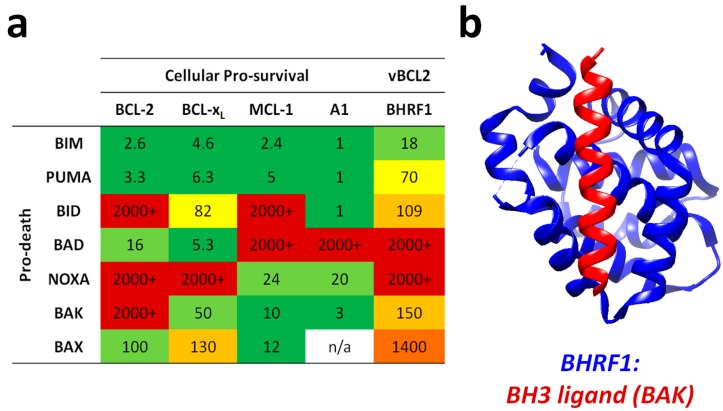
Binding specificities and affinities of BCL-2 family members and EBV BHRF1. (**a**) Interactions between pro-survival and pro-death BCL-2 family members. Reported as K_d_ in nM as determined by surface plasmon resonance (BCL-2, BCL-x_L_ and MCL-1) or isothermal calorimetry. Sources were: BCL-2 and BCL-x_L_ [144,145,146], MCL-1 [144], A1 [147] and BHRF1 [124,133]. Colour coding was applied as follows: green 1–10 nM, pale green 11–50 nM, yellow 51–100 nM, pale orange 101–1000 nM, orange 1001–2000 nM, red 2000–100,000 nM. (**b**) Ribbon structure representation of BHRF1 (blue) bound to the BH3 domain of BAK (red). This graphic was prepared using the UCSF Chimera software package (developed by the Resource for Biocomputing, Visualization, and Informatics at the University of California, San Francisco [148], supported by NIGMS P41-GM103311) using pdb accession code 2XPX [133].

The mechanism by which BHRF1 confers apoptosis protection is through binding and sequestration of cellular pro-apoptotic proteins. BHRF1 can bind to the cellular BH3-only proteins BIM, PUMA and BID and the apoptosis effector (or executioner) protein, BAK [124] (see Figure 3). One report attributes all apoptosis protection to the ability of BHRF1 to bind BIM [134,135], however work from our laboratory has shown that BHRF1 can also protect in the absence of BIM (Fitzsimmons et al. manuscript in preparation). By comparison to BHRF1, BALF1 is much less well characterised. BALF1 was identified as a BCL-2 homologue by predicted structural similarity to known vBCL-2s, and though it has been shown to modulate apoptosis, it remains controversial as to whether it promotes or inhibits cell death [130,143].

### 2.8. Non-Coding RNAs

A number of viral non-coding RNAs are expressed during transformation. Some of these transcripts are reported to regulate cell death during transformation and latency; however, their functions are less well understood than those of the latent proteins. The Epstein–Barr virus encoded RNAs (EBERs) are two highly transcribed small (~170 nt) nuclear RNAs (EBER1 and EBER2) [23,149] whose function(s) remain somewhat enigmatic. The EBERs are abundantly expressed in established LCLs but they are not detectable until around 72 h after infection of primary B cells [150]. EBV also encodes around 40 mature microRNAs (miRs/miRNAs), derived from 25 precursor (pre-miRNA) transcripts [151,152,153,154]. MicroRNAs selectively bind to and inhibit mRNAs by causing transcript instability, degradation, or impaired translation. Similar to cellular miRNAs, EBV encoded pre-miRNAs are processed by Drosher and Dicer into mature miRNAs and incorporated into the RISC complex, which then binds the target transcript to which the miRNA confers specificity by virtue of its 6 nt ‘seed sequence’ (reviewed in [155,156]). The EBV miRNAs can be divided into two families, according to their position within the viral genome. The BHRF1 miRNAs reside either side of the BHRF1 ORF, whereas the BART family of miRNAs are derived from the *Bam*HI A rightward transcripts (BARTs). Despite abundant BHRF1 and BART primary transcript expression at early time points during transformation, processing of their derivative miRNAs is delayed, peaking at around 72–120 h PI [24].

#### 2.8.1. EBERs

The EBERs are ubiquitously expressed from their own viral promoters in all forms of latent infection. There have been conflicting reports regarding the ability of EBER-KO recombinant viruses to transform B cells, although it should be noted that no previous studies have found the EBERs to be essential for transformation. Interestingly, different groups have used different strains of EBV in their experiments, suggesting that the role of the EBERs in transformation may be context dependent and influenced by the other co-expressed viral proteins. Swaminathan et al. used the transformation incompetent P3HR1 virus strain, which harbours a large genomic deletion spanning the EBNA2 locus, and recombined it with a large fragment of the prototypic B95.8 EBV that was either wt for or lacked the EBER locus (lacking both EBER1 and EBER2). The resulting recombinant wt or EBER-KO EBVs were both transformation competent [157] and were found to give rise to LCLs that grew at the same rate as wt EBV-derived LCLs [158]. However, an alternative EBER-KO virus made in the Akata virus strain was found to be less than 50% as efficient at transforming B cells than wt virus and the resulting LCLs exhibited a growth impairment in limiting dilution assays compared to wt comparators [159]. When expression of the individual EBERs was restored in this background, it was found that an EBER2 knock-in (KI) virus behaved like wt Akata virus, but the EBER1-KI (lacking EBER2) virus was as impaired as the double EBER-KO, suggesting that only EBER2 is important for transformation and that EBERs play distinct, non-overlapping roles in this process [160]. Recently however, it has been shown that EBER-KO viruses (both double and single KOs) made in the B95.8 background could transform B cells with equal efficiency to wt virus [161]. It may be relevant that this study used adult PBMCs as a source of B cells, whereas the Akata-derived virus studies were carried out on cord blood-derived cells. Interestingly, the B95.8 study identified two apoptosis-related genes as EBER targets by microarray analyses. Deletion of EBER1 led to a 6-fold downregulation of GAS2, whilst EBER2-KO cells showed around a 40-fold reduction in SASH1 [161]. Both GAS2 and SASH1 are reported to be downstream targets of Caspase 3 and have been shown to contribute to apoptosis induction [162,163]. Somewhat counterintuitively, this suggests that EBERs may prime LCLs for cell death, although further mechanistic studies have yet to be published.

#### 2.8.2. BHRF1 microRNAs

The BHRF1 family of miRNAs consists of one miRNA encoded upstream (miR-BHRF1-1) and two miRNAs encoded downstream (miR-BHRF1-2 and miR-BHRF1-3) of the BHRF1 ORF. During viral transformation of B cells, the BHRF1 miRNAs are processed from the polycistronic Wp and Cp-driven latent transcripts that encode the EBNA proteins and latent BHRF1 protein [164,165,166]. EBV genomes deleted for individual miR-BHRF1s show a mild impairment in transformation compared to wt controls and the contribution of these miRNAs was found to be cumulative. Viruses lacking miR-BHRF1-2 (Δ2) and -3 (Δ3) were the most transformation deficient, whereas viruses deleted for miR-BHRF1-1 (Δ1) transformed B cells almost as efficiently as wt EBV [167]. Accordingly, when all three BHRF1 miRNAs were deleted, the resulting Δ123 viruses showed a marked transformation defect, which was attributed to increased apoptotic cell death early after infection. Additionally, the resultant LCLs exhibited reduced S-phase entry and proliferated less well than controls [168,169]. In humanised NSG mice, animals infected with the Δ123 virus displayed lower viral loads at early time points after infection compared to mice infected with wt virus, but there was no difference in tumorigenic potential between Δ123 and wt EBV [170]. Interestingly, it was shown that ΔmiR-BHRF1 LCLs exhibit lower expression levels of BHRF1 transcripts and protein as well as other latent transcripts [31,168], indicating that at least some of the effect of miR-BHRF1 deletions were indirect. Indeed, whilst EBV lacking BHRF1 protein transformed around 50% as efficiently as wt controls, viruses lacking both BHRF1 protein and miRNAs were incapable of transforming B cells [31,129]. These results suggest that the BHRF1 miRNAs and BHRF1 protein function cooperatively to control cell cycle entry and apoptosis during primary infection.

Other than regulating the expression of the pro-survival, BHRF1 protein, it is also somewhat unclear how the BHRF1 miRNAs might further regulate apoptosis. This is, in part, because it can be difficult to experimentally confirm bona fide miRNA targets and because viral miRNAs sometimes display unique functional features compared to their cellular counterparts, such as tolerating ‘bulges’ in their mRNA seed region binding sites [165,171] (and reviewed in [172]). What is known is that EBV miRNAs bind and may contribute to the regulation of hundreds of viral and cellular transcripts, many of which have been ascribed roles in cell survival. As well as being primarily responsible for the regulation of BHRF1 protein, miR-BHRF1-2 has also been found by luciferase reporter assays to inhibit expression of the apoptosis-related genes, BACH1 and KDM4B [165], whilst miR-BHRF1-3 has been found to downregulate PTEN protein, thereby reducing cell cycle progression and, through the PI3K/Akt pathway, apoptosis [31]. Interestingly, although PTEN and BACH1 are both pro-apoptotic [173,174], KDM4B (also called JMJD2B) is a gene induced by p53 that can repress the induction of select p53 targets including p21, PIG3 and PUMA and thereby suppress cell cycle arrest and cell death [175]. This suggests that BHRF1 miRNAs might function to modulate the cell death response.

#### 2.8.3. BART microRNAs

The *BamHI* A region, which is transcriptionally active in several EBV-associated malignancies, has long been known to give rise to a complex variety of transcripts driven from two promoters (P1 and P2) [176,177,178,179]. It took until 2004 to discover that introns from these transcripts give rise to microRNAs [20]. It had previously been proposed that these transcripts encoded proteins [180,181,182], but whilst they could be transcribed and investigated in vitro, there was little evidence for the existence of *BamHI* A-derived proteins in EBV-positive cell lines [183,184]. The BART miRNAs are reported to regulate a number of cell survival and growth-related transcripts. However, the majority of studies linking BART miRNAs to survival were carried out in nasopharyngeal carcinoma (NPC) where they are highly expressed. Although the BART miRNAs are expressed at lower levels in LCLs compared to epithelial cells, they still number thousands of copies per cell in total, and are consistently detected in all types of latency [166]. Interestingly, the expression of individual BART miRNAs exhibits hierarchy, and the less abundant miRNAs are only present at tens of copies per cell in LCLs [24,165].

The prototypic EBV strain, B95.8, from which many of the widely used recombinant EBVs are derived, harbours a large (12 kbp) deletion compared to other EBV isolates [185,186], and consequently lacks most of the BART microRNAs. The readiness of the B95.8 strain and its recombinant derivatives to transform resting B cells suggests that the BART miRNAs are dispensable for transformation. Accordingly, Seto et al. found that a recombinant EBV lacking both BART and BHRF1 miRNAs was equally as efficient in transformation assays as a virus deficient in only BHRF1 miRNAs [169]. However, another study found that two B95.8 derivative viruses that had been ‘repaired’ for the BART miRNA sequences using different strategies both caused more efficient outgrowth of B cells PI. Interestingly, there was greater variation between the two different miR-BART repaired recombinant virus strains than between the B95.8 strain and the less efficient repaired strain [187].

In order to identify targets of EBV miRNAs, Dolken et al. carried out immunoprecipitation of RISC complexes (containing miRNAs and their targets) in two EBV-infected BL cell lines and then identified the target mRNA using microarray analyses. They showed that miR-BART3 and miR-BART16 downregulated IPO7 and TOM22 transcripts, respectively [188], both of which encode transport proteins and are reported to have pro-apoptotic functions [189,190]. Another group used a similar technique to investigate the cellular targets of EBV-encoded miRNA in Latency III BL cells (expressing all of the miR-BARTs), but first crosslinked the RISC complexes and RNA species together by UV-irradiation [191]. In this study, Riley et al. identified 132 apoptosis-associated genes as miR-BART targets, notably: the previously reported PTEN, IPO7 and TOM22, in addition to the EBV oncogenes, LMP1 and BHRF1; multiple pro-death BCL-2 family members, PUMA, BAK, BID, PMAIP1 (NOXA), BIM, and pro-survival MCL1; as well as CASP2, CASP3 and MYC. Only a small subset of these targets was validated in luciferase reporter assays [192]. Finally, a third group used an alternative method to crosslink miRNAs and their targets together in B95.8-derived LCLs (expressing only five BART miRNAs), followed by RNA sequencing to identify the targets. They also reported a significant enrichment among their hits for genes that can directly or indirectly influence cell survival including MCL1; the apoptosome component, APAF1; and the miRNA processing factor, DICER [165].

The use of RNA sequencing technologies has also led to the identification of a large number of novel EBV transcripts in latently infected cells [193], some of which are likely to turn out to be bona fide, functional ncRNAs [194]. Therefore, it is possible that the repertoire of EBV-encoded ncRNAs that regulate cell survival may expand in the near future.

## 3. Lytic Cycle Genes and Transformation

Although EBV is usually found to be latent in infected cells both in vivo and in vitro, the virus must periodically enter into the lytic cycle to generate infectious viral progeny to infect new cells within the host and thereby guarantee persistence and dissemination into new hosts. In vivo, the lytic cycle is thought to occur in terminally differentiated plasma cells and epithelial cells (reviewed in [195]). In vitro, replication occurs sporadically in a small population of cells in latent LCLs and tumour cell lines. Although the frequency and rate of lytic cycle activation can vary between cell lines and types, usually around 1–5% of cells are found to express the lytic cycle marker gene, BZLF1, in LCL cultures at any given time [196]. The lytic cycle is initiated by expression of BZLF1 and BRLF1, the ‘immediate early’ lytic genes, and then proceeds in two further phases of virus gene expression. BZLF1 binds and activates promoters containing Z-response elements (ZREs) [197,198], and seems to preferentially bind ZREs that are highly methylated [199,200]. BRLF1 can enhance transcription directly, by binding DNA at GC-rich promoter sequences to activate transcription [201], or indirectly, where DNA-binding is not necessary [202,203]. These transcription factors stimulate the expression of a second wave of lytic-associated viral genes, known as the early lytic genes. Early genes include those required for viral DNA synthesis, inhibition of apoptosis and immune evasion. Once the viral DNA has replicated, the late lytic genes are expressed, many of which encode structural or packaging elements of the virus.

The lytic cycle is also closely linked with cell death since the thousands of EBV particles that are produced eventually cause any cell undergoing lytic replication to lyse, releasing infectious virus [204,205]. Conversely, EBV must also ensure that cell death is not triggered too early after lytic cycle activation to allow time for progeny to be synthesised and packaged efficiently. It is unsurprising then, that EBV lytic genes have been ascribed both pro- and anti-apoptotic functions. For example, BZLF1 indirectly induces cell death via inhibition of the NF-κB family protein, p65 [206] and downregulation of CD74 [207]. Conversely, the viral BCL-2 protein, BHRF1, has been shown to directly block BZLF1 toxicity, which has the dual consequences of blocking apoptosis and contributing to the evasion of NK cell recognition and killing of BZLF1-expressing EBV-positive B cells [208]. Another group found that induction of EBV-positive cell lines into the lytic cycle also induced cell death in cell cultures. Interestingly however, the lytic BZLF1-positive cells were resistant to death compared to the cells that remained latent, further suggesting that EBV expresses potent anti-apoptotic genes upon lytic reactivation. Furthermore, cells that were treated with acyclovir (ACV) to specifically block late lytic cycle EBV gene expression exhibited an increased propensity to undergo apoptosis, suggesting that both early and late lytic genes contribute to cell survival during virus replication [209]. The BH3-only BCL-2 family protein, BIM, has also been shown to be downregulated in a two-step fashion during lytic cycle, suggesting that it is a common target for both early and late EBV lytic genes [210].

In primary B cells, a number of lytic-cycle-associated EBV genes have been shown to be transcribed early during transformation (1–4 days PI.) including BZLF1, BRLF1, BCRF1 (viral IL-10), BALF1, BHRF1 and BMRF1 in the absence of any production of infectious virions [129,199,211,212]. Conversely, inhibition of apoptosis by miR-BART20-5p has been shown to indirectly inhibit the lytic cycle, suggesting that latent apoptosis regulatory genes may also play a role in EBV replication [213]. There also remain a number of EBV genes that are assigned as ‘lytic’ about which almost nothing is known. LF3, for example, is classified as a lytic gene since it is robustly transcriptionally upregulated during the lytic cycle [214]. However, RNA-sequencing and quantitative PCR studies showed that LF3 is also highly transcribed in largely latent cell lines [33,193]. Together, these studies demonstrate the interplay between apoptosis and the EBV lytic cycle and raise the possibilities that (1) the definition between latency and lytic cycle may be less well defined than current dogma dictates, (2) some lytic genes may regulate cell death and (3) poorly described EBV genes may contribute to cell death regulation during latency as well as lytic cycle. Further work is required to better understand the role of lytic cycle genes in the regulation of cell death during transformation and in malignancy in order to determine whether these processes might be useful therapeutic targets for EBV-associated diseases.

## 4. Cooperative Cell Death Inhibition by EBV

### 4.1. Counteracting the DNA Damage Response

A number of studies have provided insight into the interplay between latent proteins and host cell survival pathways through analyses of early infection (the first 7–10 days PI.) during the establishment of latency (Figure 2). EBV infected B cells begin to proliferate rapidly at around 4 days PI. [32,215,216,217], when the Wp transcript-encoded EBNA and BHRF1 proteins are maximally expressed [19,25,30,31]. Coincident with entry into cell cycle, activated lymphoblasts initiate the DNA damage response, including robust upregulation of p53 and related pro-apoptotic genes [216,218,219,220]. Many oncogenic viruses (including SV40, HPV and adenovirus) must directly inactivate p53 in order to transform cells (reviewed [221]), yet in the case of EBV, p53 signalling remains intact in established LCLs [218]. However, DNA damage signalling and p53-induced apoptosis must be countered in order for EBV to transform resting B cells [222]. Furthermore, CRISPR screens have revealed that the p53 inhibitors, MDM2 and MDM4, remain critical for the ongoing survival of established LCLs [105]. Despite markers of DNA damage including γH2AX and chromosomal abnormalities appearing within the first seven days following infection [216,223], few cells are found to undergo apoptosis at these early time points [32,215,217]. It is hypothesised that EBNA3C is largely responsible for preventing p53-driven apoptosis during early time points PI. Since it has been reported to bind and/or stabilise several p53 inhibitors including Gemin3, Aurora B kinase, ING4, ING5 and MDM2 [70,72,224,225,226] and also to repress the p53 activator p14^ARF^ [66]. Interestingly, inhibition of p53-mediated apoptosis also remains essential long-term as a CRISPR screen has recently shown that MDM2 and MDM4 are critical for the survival of established LCLs [105].

**Figure 4:**
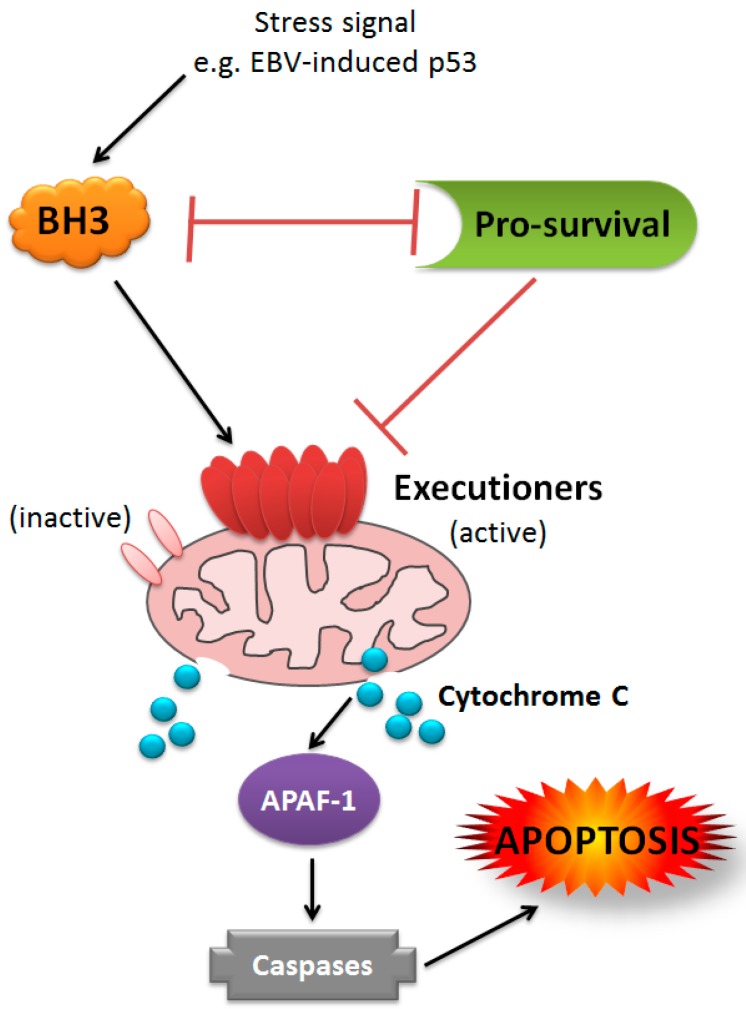
Model of BCL-2 family-mediated intrinsic apoptosis. Schematic of interactions among different classes of BCL-2 family members in the intrinsic apoptosis pathway. Pro-survival, BCL-2-like proteins, including BHRF1 (green), inhibit both classes of pro-death proteins, though the affinities and specificities for these binding partners vary. BH3-only, pro-death proteins (orange) inhibit pro-survival BCL-2s through reciprocal binding and some can directly activate executioner pro-death BCL-2 homologues (only BIM, PUMA and t-BID are able to perform direct activation). The executioner BCL-2s, BAK and BAX exist as inactive monomers until activated by BH3-only proteins or disinhibited by pro-survival BCL-2s. Upon activation, BAK and/or BAX multimerise to form pores in the mitochondrial outer membrane, causing the release of cytochrome C (and other pro-apoptotic factors), which then activate APAF-1, leading to the assembly of the apoptosome, caspase cleavage and consequent cell destruction.

Following activation of p53, cell death proceeds through the BCL-2 family/intrinsic pathway to apoptosis, via p53-induced upregulation of the BH3-only proteins PUMA and NOXA. These BH3-only proteins initiate cell death by binding to the BCL-2 pro-survival proteins, thereby releasing the executioners of apoptosis BAX and BAK. These proteins form pores in the mitochondrial outer membrane (MOMP) which commits the cell to apoptosis. BAK and BAX can also be directly activated by the BH3-only proteins, BIM, tBID and PUMA, though only PUMA is upregulated by p53 activation (reviewed in [227]) (see Figure 4). Many human viruses have evolved strategies to interfere with the intrinsic apoptotic pathway (reviewed in [228]). EBV itself encodes two multi-domain BCL-2 homologues, BHRF1 and BALF1, as discussed earlier. BHRF1 may contribute to the blockade of p53-dependent DDR signalling as it is able to bind and inhibit pro-apoptotic PUMA, [133] and can efficiently inhibit DNA-damage-induced cell death in the absence of any other EBV genes (Fitzsimmons et al. in preparation). LMP1 has been shown to block apoptosis through upregulation of cellular BCL-2 pro-survival proteins [98,99,101,102]. As discussed throughout this review, EBNA1, EBNA2, the EBNA3s, and both families of EBV miRNAs, also regulate a variety of different BCL-2 family members and associated intrinsic apoptosis regulatory elements. Consistent with this complex regulation by multiple EBV genes, a recent study showed that EBV-infected lymphoblasts and LCLs do not rely on any single BCL-2 family member for survival. Instead, the sensitivity of EBV-infected cells to BH3-only proteins changes throughout the process of growth transformation [32].

### 4.2. Cooperation in Transformation

The in vitro growth transformation of B cells by EBV requires that the virus efficiently activates the cell cycle such that the infected cells will proliferate continually, but also necessitates robust and comprehensive inhibition of cell death [229]. Studies of transformed cells and on the process of transformation have been instrumental in revealing how the latent genes of EBV operate a multi-faceted, fail-safe system to regulate key cell fate signalling pathways. For example, multiple BCL-2 family members are known to be regulated by latent EBV genes and many are targeted by more than one viral gene product. Despite this belt-and-braces approach to overcoming cell death, EBV-driven transformation is not efficient: only a fraction of infected cells survive to become continuously proliferating LCLs [230,231]. This may be because EBV-mediated rewiring of the B cell proliferation machinery concomitantly triggers tumour suppressor, cell death and anti-viral pathways, meaning that LCLs remain constantly ‘primed’ for cell death. However, more likely it demonstrates how evolution has selected for a carefully orchestrated balance between the agonistic and antagonistic functions of the various multifunctional latent genes that allow EBV to respond dynamically to different stimuli and trigger or block cell death efficiently, depending on circumstance. Detailed understanding of the combinatorial and/or redundant effects of EBV genes in apoptosis regulation in a given disease setting is imperative to inform therapeutic treatments. To this end, the recent development of new methods such as CRISPR/Cas9 to quickly and precisely genetically modify the EBV genome within infected cells [232] will be central for dissecting the cooperation between EBV genes in cell death regulation.

### 4.3. Cooperative Inhibition of Apoptosis in Malignancy

EBV is known to contribute to various forms of malignancy of lymphoid and epithelial cell origin, together accounting for around 200,000 new cancer diagnoses annually [4]. Although the role of the virus is not fully understood in these EBV-associated cancers, there is a large body of evidence to suggest that the virus contributes to the inhibition of cell death in many cases. Whilst the transformation of resting B cells into continuously proliferating LCLs is a useful model for EBV infection, the pattern of latent gene expression seen in LCLs (Latency III) is rarely seen in EBV-positive tumours. If fact, it is usually only detected in patients who are immunocompromised, for example in EBV-driven post-transplant lymphoproliferative disease (PTLD) or in HIV-positive individuals [1]. In these cases, where the immunological imperative for restricted latency is relieved, the cooperative contribution of the Latency III genes is likely to largely reflect those seen in LCLs. Accordingly, although relatively few mechanistic studies exist on EBV-mediated apoptosis inhibition in PTLD, there are reports that key genes implicated in cell death regulation in LCLs, such as Syk, IL10 and BIM, are also deregulated in this malignancy [233,234,235]. In all other known EBV-associated malignancies, EBV usually adopts more restricted and less immunogenic latencies that vary among different EBV-associated cancers (Figure 1). This means that the most abundant or functionally important EBV targets for therapy may differ among different EBV-associated malignancies. Another important consideration in this regard is that there is evidence that EBV genes work together to cooperatively modulate cell survival pathways in diverse EBV-associated cancers, even in BLs, which exhibit the most restricted form of EBV latency (Latency I). Whilst this raises the possibility that targeting a single EBV protein in malignancy may be ineffective, it does suggest that therapies which disrupt viral hijacking of these critical pathways might be of clinical benefit across multiple EBV malignancies. Here, we review in detail the role of EBV genes in the cooperative inhibition of apoptosis in BL.

## 5. Burkitt Lymphoma (BL)

The frequency and geographical incidence of BL in malarial endemic regions of sub-Saharan Africa is consistent with an infectious aetiology and this led to the identification of BL as the first cancer to be associated with EBV infection. Accordingly, EBV was found to reside in more than 95% of BLs in high incidence or endemic BL (eBL) areas. BL also occurs worldwide at a lower incidence, and in these cases, known as sporadic BLs (spBL), EBV is found in 15–85% of tumours, varying by geographical region [236,237,238,239].

### 5.1. c-MYC

Common to all cases of BL is the hallmark reciprocal translocation between the *c-MYC* oncogene and a constitutively active immunoglobulin (Ig) gene promoter/enhancer [240,241,242]. This unchecked overexpression of the c-MYC protein reprograms cells for maximum proliferative capacity [243,244,245]. Conversely however, *c-MYC* also sensitises cells to apoptosis under conditions of stress [246]. Therefore, in this tumour setting, constitutive c-MYC expression circumvents the requirement for EBV to drive proliferation, but it does place an imperative on EBV to block cellular apoptosis during oncogenesis. One well-characterised pro-apoptotic pathway activated by *c-MYC* is the ARF-MDM2-p53-axis. Accordingly, around 30% of BL biopsies [247] and 70% of BL cell lines have mutated p53 sequences [248,249,250]. Furthermore, in BLs where p53 has a wild type sequence, p14^ARF^ or MDM2 is frequently altered in their expression in order to circumvent p53-dependent cell death [251,252]. Additionally, deregulation of the downstream BCL-2 family pathway accelerates MYC-induced lymphomagenesis in the *Eμ-Myc* mouse model of BL, which carries a *c-MYC;* IgM heavy chain enhancer transgene. Blocking the intrinsic apoptotic pathway through genetic deletion of pro-apoptotic BCL-2 family genes or constitutive overexpression of BCL-2 pro-survival proteins can all accelerate lymphoma onset in this mouse model [253,254,255,256,257,258,259]. Furthermore, silencing of the pro-apoptotic BH3-only genes PUMA and BIM has been described in human BL tumours [260,261].

Interestingly, *c-MYC* itself is frequently mutated in BL, and this is hypothesised to reflect a need to avoid apoptosis activation whilst retaining or enhancing proliferative capability [262,263,264,265]. Indeed, one *c-MYC* mutant that commonly occurs in BL has been reported to be incapable of inducing BIM and consequently cannot trigger cells into apoptosis [266]. Outside of the p53 and BCL-2 pathways, mutations in other pro-survival signalling pathways may also play a role in BL. Recent genome-wide studies have identified the transcription factor E2A (TCF-3), ID3, and cyclin D3 as frequently mutated in BL biopsies and cell lines [262,265,267,268,269]. Interestingly, these targets all contribute to a third survival axis, which is ultimately co-ordinated by PI3K—hence several different diversion mechanisms may contribute to apoptosis escape in BL (reviewed in [270]).

### 5.2. The Contribution of EBV

In EBV-positive BL, the virus is present as multiple viral episomes in every tumour cell and is maintained indefinitely, suggesting a selective advantage associated with virus retention. Treating BL cells with a dominant negative derivative of the viral episome maintenance protein, EBNA1 (dnEBNA1), enforces loss of viral genomes and induces apoptosis, suggesting that EBV is essential for the continued survival of these BL cells [271,272]. Incidences of EBV-negative BL do occur, albeit with lower frequency, and interestingly analyses have revealed EBV-positive and -negative BLs to be genetically distinct; differing in the number of somatic mutations, frequency of chromosomal copy number changes and the precise *c-MYC* translocations [262,273,274,275,276,277]. This suggests that EBV-positive and -negative BLs arise via slightly different pathogenic routes and furthermore, that the role of EBV in BL needs to be examined in the context of virus positive lymphomas. Given that subversion of apoptosis is an essential step in *MYC*-driven lymphomagenesis [278], it has long been suggested that EBV may provide protection against intrinsic apoptosis in the established tumour.

### 5.3. Restricted EBV Latency in BL

#### 5.3.1. Latency I

In 85% of eBLs, the virus expresses only EBNA1, the EBER transcripts and the miR-BARTs, collectively termed Latency I (Figure 1). EBNA1, EBERs and miR-BARTs are found to be expressed in all EBV-associated malignancies, but when expressed in the absence of the other latent proteins, this Latency I pattern is the most restricted form of viral gene expression found in EBV-associated cancers. Thus, the Latency I gene products constitute the minimum ongoing contribution of EBV to cancer. The nature of this ongoing viral contribution to BL has been much discussed, but remains somewhat controversial. In 1994, a report that spontaneous loss of EBV from a sporadic BL cell line called Akata-BL increased the sensitivity of the cells to apoptosis, impaired their growth and rendered them non-tumorigenic in a mouse model, provided compelling evidence that the virus remains critical for the survival of BL cells. Furthermore, it demonstrated for the first time a system in which isogenic cells that contained or had ‘lost’ the virus could be directly compared [279]. This group later showed that the ‘EBV-loss’ phenotype could be reversed by restoring the virus genome—a key result which directly implicated the virus as a protective agent in BL [280]. A different group used hydroxyurea to eliminate the viral genome from two BL cell lines; Mutu-BL and Akata-BL, but found that only Akata-BL-derived EBV-loss cells showed an increase in apoptosis sensitivity [281]. An alternative strategy was used by the Sugden group, who found that enforced loss of EBV genomes using dnEBNA1 consistently induces apoptosis in BL cells [271,272,282,283,284]. Although this approach convincingly demonstrates the complete dependency of BL cells on EBV for their ongoing survival, the toxic nature of the phenotype makes it difficult to isolate EBV-loss clones for comparison with EBV-positive cells, as was previously done. Recently we reported a large-scale study investigating the rates of spontaneous EBV loss from BL cell lines and found that, whilst the phenomenon is rare (loss clones comprised just 3% of those screened), it was consistently associated with an increase in apoptosis sensitivity. Importantly, and consistent with other laboratories, we showed that reinfection of EBV-loss BL cells with recombinant EBV and the establishment of a Latency I infection could reverse the apoptosis-sensitive phenotype [280,285,286].

The mechanism of apoptosis protection by EBV in Latency I BL appears to be cooperative and to exhibit redundancy. Whilst EBERs are reported to promote survival by upregulating IL10 in some clonal EBV-loss variants of Akata-BL [287], we and others have found that neither EBNA1 nor EBERs alone can restore apoptosis protection to EBV-loss BLs [285,286,288]. Interestingly however, the Sample group, who first showed that EBERs alone could not protect BL cells, did find that BL cells expressing both EBNA1 and EBERs were tumorigenic in vivo, although to a lesser extent than BL cells infected with wt EBV [288]. Additionally, whilst we have found that expression of miR-BARTs alone could not confer any protection to EBV-loss BL cells [285], miR-BARTs, when expressed in combination with EBNA1, have been shown to reduce dnEBNA1-induced cell death [187]. Furthermore, when EBV-loss cells were reinfected with a recombinant, Latency I-restricted EBV that lacks many of the miR-BARTs, they exhibited a moderate, but significant reduction in apoptosis resistance [285]. As mentioned previously (see Section 2.6), LMP2A can regulate important cell death pathways. Accordingly, ectopic expression of LMP2A has been shown to protect some BL cell lines from apoptosis [121,289], and can enhance tumorigenicity in a mouse model [290,291]. However, whilst LMP2 transcripts are detectable in BL tumour material [292,293,294], absolute quantitation has shown that the abundance of these transcripts in Latency I cells is low compared to those found in other forms of latency [33,193]. Additionally, LMP2A protein expression is a consistent feature of LCLs and Latency II malignancies, however is rarely detected in BL cells [33,292]. Therefore, it has been extensively demonstrated that optimal cell death inhibition in Latency I BL can only be conferred by the presence and cooperation of all of the Latency I-associated EBV genes. Although the precise mechanisms by which each Latency I gene product inhibits cell death in BL has not been fully elucidated, the collective presence of all Latency I products can block the induction of both BIM and PUMA following apoptotic challenge [285]. BIM and PUMA translation is likely inhibited by miR-BARTs as their 3′-UTRs contain multiple predicted binding sites for a variety of BART miRNAs [165,192,295,296], though miR-BARTs alone are insufficient to appreciably downregulate BIM and/or PUMA in BL cells [285]. EBERs meanwhile, may contribute to the suppression of BIM and PUMA induction via activation of AKT/PI3K signalling [297], which has been shown to regulate BIM and PUMA expression at both transcriptional and post-translational level [298]. PUMA is also subject to regulation by the TGF-β pathway [299,300], which EBNA1 has been shown to modulate through downregulation of SMAD2 [82,301]. Caspase 3 expression is also reduced in both EBV-positive and miR-BART expressing Latency I BL cells compared to levels in dnEBNA1-treated control cells [187]. Therefore, it is possible that there is a third, downstream pro-apoptotic target for Latency I EBV genes. The importance of BIM and PUMA in BL cell survival has also been demonstrated by the finding that their expression is often selected against by epigenetic silencing [64,260], though further work is required to establish whether these changes occur more commonly in EBV-negative versus EBV-positive tumours. In summary, the Latency I EBV genes (EBNA1, EBERs and miR-BARTs), collectively suppress induction of pro-apoptotic BIM and PUMA via cooperative targeting of their transcription and translation in order to overcome a c-MYC-induced sensitivity to apoptosis.

#### 5.3.2. Wp-Restricted Latency

In the remaining ~15% of eBLs, EBV adopts a more extensive pattern of gene expression involving EBNA-3A, -3B and -3C, a truncated form of EBNA-LP and the BHRF1 protein, in addition to EBNA1, EBERs and miR-BARTs [19,302,303]. More recently, we have also found that two of the three BHRF1 miRNAs are also expressed in these tumours [24]. This pattern of gene expression, termed Wp-restricted Latency (Wp-BL) is imposed by a large deletion in the virus genome and is associated with marked resistance to cell death [302]. The exact size of the deletion varies between tumour samples, but always encompasses the coding region of the EBNA2 gene, placing BHRF1 adjacent to the promoter-encoding Wp repeat region [303]. This promoter, which is silent in Latency I BL, drives high levels of BHRF1 expression and also transcribes the EBNA proteins [19]. EBNA-3A and -3C are known to cooperatively downregulate BIM and p16^INK4a^ through epigenetic silencing leading to reduced sensitivity to cell death [59,64,65,304]. However, it has been shown that short-term expression of EBNA3s, either individually or in combination, cannot protect BL cells to the same extent as Wp-restricted latency [19]. Wp-BLs also express a variant of EBNA-LP that is truncated (t-EBNA-LP) compared to the form expressed in Latency III as a result of the characteristic EBV genomic deletion. Interestingly, this t-EBNA-LP has also been shown to contribute to cell survival via inhibition of protein phosphatase 2A (PP2A), a function apparently specific to the truncated form and not shared by the full length EBNA-LP [305]. BHRF1, by virtue of the fact that it is a viral BCL-2 homologue, can efficiently inhibit cell death even in the absence of other EBV genes via binding to and inhibition of cellular pro-apoptotic proteins BIM, PUMA, BID and BAK [19,133]. The propensity of BHRF1 to bind particular pro-death cellular proteins may vary under different conditions of cell stress (unpublished data from our lab). Therefore, EBV likely exhibits potent cell death inhibition in Wp-BL through a cooperative mechanism involving the EBNA3, t-EBNA-LP and BHRF1 proteins and possibly miR-BHRF1s, though the role of the miRNAs may be an indirect effect on regulation of BHRF1 itself [31].

## 6. Future Perspectives

Cooperation among EBV genes to inhibit cell death is clearly important for both growth transformation and the pathogenesis of BL; however, relatively little is known about how EBV genes cooperate outside of these models. There is evidence in the literature to suggest that EBV targets common pathways in different disease settings. For example, EBV is now known to be an etiologic factor in around 9% of gastric carcinomas (GC). As in other EBV-associated cancers, the virus is present as a latent infection in these tumours, although there are conflicting reports as to whether it establishes Latency I or II infection (the latter involves expression of LMP1 and LMP2 proteins in addition to the Latency I genes) [306]. Like BL, EBV-positive and -negative cases of GC are genetically distinct. EBV-positive GCs can be distinguished by marked hypermethylation of genes, including universal silencing of p16^INK4A^, and frequent characteristic mutations in PIK3CA, ARID1A and BCOR genes [306,307]. The silencing of p16^INK4A^ and the 80% incidence of mutations in the PI3K family gene, PIK3CA, suggest that the same key signalling pathways identified in BL and LCLs are also important in GC pathogenesis. Similarly, in the setting of NPC, where EBV establishes a Latency II infection, the *INK4A* locus (encoding p16^INK4A^ and p14^ARF^) is commonly mutated or silenced. Therefore, possible cooperation between EBV genes to regulate key survival signalling pathways, in particular BCL-2 family signalling, PI3K signalling and the ARF-MDM2-p53-axis, in EBV-associated cancers warrants further investigation.

Whilst it is clear that the known latent genes of EBV make a considerable contribution to the apoptosis phenotype of EBV-associated cancers, the roles of other EBV-encoded genes in this regard require further attention. Lytic-cycle-associated transcripts can be readily detected in EBV-positive cancers [33,193,267,306,308] and though these are thought to originate from the small percentage of cells within the population that spontaneously undergo lytic replication, more detailed single cell analyses are required to confirm whether any of these transcripts (and where relevant, the proteins they encode) are detectable in otherwise latently infected tumour cells. For example, the expression of BHRF1 protein was initially thought to be restricted to the lytic cycle [131], however it is now known to be expressed in latently infected Wp-BL and LCLs [19]. Of note in this regard, BHRF1 transcripts have also been reported in 15–20% of otherwise latent EBV-positive GC biopsies, whilst BARF1 transcripts were detected in 40–100% of samples (6/13 and 9/9 biopsies, respectively) [309,310]. Furthermore, BALF1 has also been detected in 80% of NPC biopsies (13/16) [311]. This analysis is further complicated by the findings that BARF1 can be secreted from EBV infected cells [312] and certain EBV gene products, including BART miRNAs and LMP proteins, can be released from infected cells in exosomes, and thereby influence the survival of neighbouring cells [313] (EBV exosomes reviewed in [314]). Evidence is also emerging that novel splice variants of LMP2A (and possibly other latent genes) may be expressed during the lytic cycle or in rarely infected cell types, such as NK or T cells, raising the possibility for additional or modified apoptosis-regulating functionality by latent genes in these settings [315,316]. New techniques to genetically modify EBV genomes in situ, such as CRISPR/Cas9 [232,317], will be critical to dissect the contribution(s) of these lesser characterised EBV genes.

Whilst the evidence that EBV can regulate apoptosis is compelling, future investigations may also uncover mechanisms through which EBV can modulate additional forms of cell death. For example, the LMP1 protein has been reported to modulate autophagy by both NF-κB-dependent and -independent mechanisms [318,319] and autophagy has recently been reported to play an important role in cell survival during growth transformation of B cells [217]. Furthermore, a number of recent studies have identified viral modulators of emerging cell death processes, such as necroptosis and pyroptosis in viruses including cytomegalovirus, vaccinia virus and hepatitis C virus [320,321,322]. Whilst little is known about EBV in this regard, these findings further open up the field of viral modulation of cell death. Therefore, whilst the study of growth transformation and BL have had a profound impact on our understanding of EBV manipulation of cell survival, future work taking into account the possibility of cooperation among the full spectrum of EBV genes in regulating both well-characterised and emerging cell death pathways will be key to the development of new therapeutic approaches.

## References

[1] Rickinson A.A.E.K. (2001). Fields Virology.

[2] Epstein M.A., Achong B.G., Barr Y.M. (1964). Virus Particles in Cultured Lymphoblasts from Burkitt’s Lymphoma. Lancet.

[3] Epstein M.A., Barr Y.M., Achong B.G. (1964). A Second Virus-Carrying Tissue Culture Strain (Eb2) of Lymphoblasts from Burkitt’s Lymphoma. Pathol. Biol. (Paris).

[4] Cohen J.I., Fauci A.S., Varmus H., Nabel G.J. (2011). Epstein-Barr virus: An important vaccine target for cancer prevention. Sci. Transl. Med..

[5] Burkitt D. (1958). A sarcoma involving the jaws in African children. Br. J. Surg..

[6] Burkitt D.P., Wright D.H. (1970). Burkitt’s Lymphoma.

[7] Burkitt D. (1963). A Children’s Cancer with Geographical Limitations. Cancer Prog..

[8] Burkitt D.P. (1961). Observations on the geography of malignant lymphoma. East Afr. Med. J..

[9] Haddow A.J. (1963). An Improved Map for the Study of Burkitt’s Lymphoma Syndrome in Africa. East Afr. Med. J..

[10] Haddow A.J. (1964). Age Incidence in Burkitt’s Lymphoma Syndrome. East Afr. Med. J..

[11] Kelly G.L., Rickinson A.B. (2007). Burkitt lymphoma: Revisiting the pathogenesis of a virus-associated malignancy. Hematol. Am. Soc. Hematol. Educ. Progr..

[12] Crawford D.H., Rickinson A., Johannessen I.O. (2014). Cancer Virus: The Story of Epstein-Barr Virus.

[13] Pope J.H., Horne M.K., Scott W. (1968). Transformation of foetal human keukocytes in vitro by filtrates of a human leukaemic cell line containing herpes-like virus. Int. J. Cancer.

[14] Henle W., Diehl V., Kohn G., Zur Hausen H., Henle G. (1967). Herpes-type virus and chromosome marker in normal leukocytes after growth with irradiated Burkitt cells. Science.

[15] Lindahl T., Adams A., Bjursell G., Bornkamm G.W., Kaschka-Dierich C., Jehn U. (1976). Covalently closed circular duplex DNA of Epstein-Barr virus in a human lymphoid cell line. J. Mol. Biol..

[16] Hammerschmidt W., Sugden B. (2013). Replication of Epstein-Barr viral DNA. Cold Spring Harb. Perspect. Biol..

[17] Rowe D.T., Rowe M., Evan G.I., Wallace L.E., Farrell P.J., Rickinson A.B. (1986). Restricted expression of EBV latent genes and T-lymphocyte-detected membrane antigen in Burkitt’s lymphoma cells. EMBO J..

[18] Rowe M., Rowe D.T., Gregory C.D., Young L.S., Farrell P.J., Rupani H., Rickinson A.B. (1987). Differences in B cell growth phenotype reflect novel patterns of Epstein-Barr virus latent gene expression in Burkitt’s lymphoma cells. EMBO J..

[19] Kelly G.L., Long H.M., Stylianou J., Thomas W.A., Leese A., Bell A.I., Bornkamm G.W., Mautner J., Rickinson A.B., Rowe M. (2009). An Epstein-Barr virus anti-apoptotic protein constitutively expressed in transformed cells and implicated in burkitt lymphomagenesis: The Wp/BHRF1 link. PLoS Pathog..

[20] Pfeffer S., Zavolan M., Grasser F.A., Chien M., Russo J.J., Ju J., John B., Enright A.J., Marks D., Sander C. (2004). Identification of virus-encoded microRNAs. Science.

[21] Xing L., Kieff E. (2007). Epstein-Barr virus BHRF1 micro- and stable RNAs during latency III and after induction of replication. J. Virol..

[22] Cai X., Schafer A., Lu S., Bilello J.P., Desrosiers R.C., Edwards R., Raab-Traub N., Cullen B.R. (2006). Epstein-Barr virus microRNAs are evolutionarily conserved and differentially expressed. PLoS Pathog..

[23] Rosa M.D., Gottlieb E., Lerner M.R., Steitz J.A. (1981). Striking similarities are exhibited by two small Epstein-Barr virus-encoded ribonucleic acids and the adenovirus-associated ribonucleic acids VAI and VAII. Mol. Cell. Biol..

[24] Amoroso R., Fitzsimmons L., Thomas W.A., Kelly G.L., Rowe M., Bell A.I. (2011). Quantitative studies of Epstein-Barr virus-encoded microRNAs provide novel insights into their regulation. J. Virol..

[25] Alfieri C., Birkenbach M., Kieff E. (1991). Early events in Epstein-Barr virus infection of human B lymphocytes. Virology.

[26] Austin P.J., Flemington E., Yandava C.N., Strominger J.L., Speck S.H. (1988). Complex transcription of the Epstein-Barr virus BamHI fragment H rightward open reading frame 1 (BHRF1) in latently and lytically infected B lymphocytes. Proc. Natl. Acad. Sci. USA.

[27] Abbot S.D., Rowe M., Cadwallader K., Ricksten A., Gordon J., Wang F., Rymo L., Rickinson A.B. (1990). Epstein-Barr virus nuclear antigen 2 induces expression of the virus-encoded latent membrane protein. J. Virol..

[28] Zimber-Strobl U., Kremmer E., Grasser F., Marschall G., Laux G., Bornkamm G.W. (1993). The Epstein-Barr virus nuclear antigen 2 interacts with an EBNA2 responsive cis-element of the terminal protein 1 gene promoter. EMBO J..

[29] Schlager S., Speck S.H., Woisetschlager M. (1996). Transcription of the Epstein-Barr virus nuclear antigen 1 (EBNA1) gene occurs before induction of the BCR2 (Cp) EBNA gene promoter during the initial stages of infection in B cells. J. Virol..

[30] Shannon-Lowe C., Baldwin G., Feederle R., Bell A., Rickinson A., Delecluse H.J. (2005). Epstein-Barr virus-induced B-cell transformation: Quantitating events from virus binding to cell outgrowth. J. Gen. Virol..

[31] Bernhardt K., Haar J., Tsai M.H., Poirey R., Feederle R., Delecluse H.J. (2016). A Viral microRNA Cluster Regulates the Expression of PTEN, p27 and of a bcl-2 Homolog. PLoS Pathog..

[32] Price A.M., Tourigny J.P., Forte E., Salinas R.E., Dave S.S., Luftig M.A. (2012). Analysis of Epstein-Barr virus-regulated host gene expression changes through primary B-cell outgrowth reveals delayed kinetics of latent membrane protein 1-mediated NF-κB activation. J. Virol..

[33] Tierney R.J., Shannon-Lowe C.D., Fitzsimmons L., Bell A.I., Rowe M. (2015). Unexpected patterns of Epstein-Barr virus transcription revealed by a High throughput PCR array for absolute quantification of viral mRNA. Virology.

[34] Hofelmayr H., Strobl L.J., Marschall G., Bornkamm G.W., Zimber-Strobl U. (2001). Activated Notch1 can transiently substitute for EBNA2 in the maintenance of proliferation of LMP1-expressing immortalized B cells. J. Virol..

[35] Strobl L.J., Hofelmayr H., Marschall G., Brielmeier M., Bornkamm G.W., Zimber-Strobl U. (2000). Activated Notch1 modulates gene expression in B cells similarly to Epstein-Barr viral nuclear antigen 2. J. Virol..

[36] Sakai T., Taniguchi Y., Tamura K., Minoguchi S., Fukuhara T., Strobl L.J., Zimber-Strobl U., Bornkamm G.W., Honjo T. (1998). Functional replacement of the intracellular region of the Notch1 receptor by Epstein-Barr virus nuclear antigen 2. J. Virol..

[37] Grossman S.R., Johannsen E., Tong X., Yalamanchili R., Kieff E. (1994). The Epstein-Barr virus nuclear antigen 2 transactivator is directed to response elements by the J kappa recombination signal binding protein. Proc. Natl. Acad. Sci. USA.

[38] Yalamanchili R., Tong X., Grossman S., Johannsen E., Mosialos G., Kieff E. (1994). Genetic and biochemical evidence that EBNA 2 interaction with a 63-kDa cellular GTG-binding protein is essential for B lymphocyte growth transformation by EBV. Virology.

[39] Zimber-Strobl U., Strobl L.J., Meitinger C., Hinrichs R., Sakai T., Furukawa T., Honjo T., Bornkamm G.W. (1994). Epstein-Barr virus nuclear antigen 2 exerts its transactivating function through interaction with recombination signal binding protein RBP-J kappa, the homologue of Drosophila Suppressor of Hairless. EMBO J..

[40] Henkel T., Ling P.D., Hayward S.D., Peterson M.G. (1994). Mediation of Epstein-Barr virus EBNA2 transactivation by recombination signal-binding protein J kappa. Science.

[41] Tierney R., Nagra J., Hutchings I., Shannon-Lowe C., Altmann M., Hammerschmidt W., Rickinson A., Bell A. (2007). Epstein-Barr virus exploits BSAP/Pax5 to achieve the B-cell specificity of its growth-transforming program. J. Virol..

[42] Lee J.M., Lee K.H., Weidner M., Osborne B.A., Hayward S.D. (2002). Epstein-Barr virus EBNA2 blocks Nur77-mediated apoptosis. Proc. Natl. Acad. Sci. USA.

[43] Lee J.M., Lee K.H., Farrell C.J., Ling P.D., Kempkes B., Park J.H., Hayward S.D. (2004). EBNA2 is required for protection of latently Epstein-Barr virus-infected B cells against specific apoptotic stimuli. J. Virol..

[44] Godoi P.H., Wilkie-Grantham R.P., Hishiki A., Sano R., Matsuzawa Y., Yanagi H., Munte C.E., Chen Y., Yao Y., Marassi F.M. (2016). Orphan Nuclear Receptor NR4A1 Binds a Novel Protein Interaction Site on Anti-apoptotic B Cell Lymphoma Gene 2 Family Proteins. J. Biol. Chem..

[45] Pegman P.M., Smith S.M., D’Souza B.N., Loughran S.T., Maier S., Kempkes B., Cahill P.A., Simmons M.J., Gelinas C., Walls D. (2006). Epstein-Barr virus nuclear antigen 2 trans-activates the cellular antiapoptotic bfl-1 gene by a CBF1/RBPJ kappa-dependent pathway. J. Virol..

[46] Campion E.M., Hakimjavadi R., Loughran S.T., Phelan S., Smith S.M., D’Souza B.N., Tierney R.J., Bell A.I., Cahill P.A., Walls D. (2014). Repression of the proapoptotic cellular BIK/NBK gene by Epstein-Barr virus antagonizes transforming growth factor beta1-induced B-cell apoptosis. J. Virol..

[47] Wood C.D., Veenstra H., Khasnis S., Gunnell A., Webb H.M., Shannon-Lowe C., Andrews S., Osborne C.S., West M.J. (2016). MYC activation and BCL2L11 silencing by a tumour virus through the large-scale reconfiguration of enhancer-promoter hubs. eLife.

[48] Sinclair A.J., Palmero I., Peters G., Farrell P.J. (1994). EBNA-2 and EBNA-LP cooperate to cause G0 to G1 transition during immortalization of resting human B lymphocytes by Epstein-Barr virus. EMBO J..

[49] Mannick J.B., Cohen J.I., Birkenbach M., Marchini A., Kieff E. (1991). The Epstein-Barr virus nuclear protein encoded by the leader of the EBNA RNAs is important in B-lymphocyte transformation. J. Virol..

[50] Hammerschmidt W., Sugden B. (1989). Genetic analysis of immortalizing functions of Epstein-Barr virus in human B lymphocytes. Nature.

[51] Tierney R.J., Kao K.Y., Nagra J.K., Rickinson A.B. (2011). Epstein-Barr virus BamHI W repeat number limits EBNA2/EBNA-LP coexpression in newly infected B cells and the efficiency of B-cell transformation: A rationale for the multiple W repeats in wild-type virus strains. J. Virol..

[52] Kashuba E., Yurchenko M., Szirak K., Stahl J., Klein G., Szekely L. (2005). Epstein-Barr virus-encoded EBNA-5 binds to Epstein-Barr virus-induced Fte1/S3a protein. Exp. Cell Res..

[53] Kashuba E., Mattsson K., Pokrovskaja K., Kiss C., Protopopova M., Ehlin-Henriksson B., Klein G., Szekely L. (2003). EBV-encoded EBNA-5 associates with P14ARF in extranucleolar inclusions and prolongs the survival of P14ARF-expressing cells. Int. J. Cancer.

[54] Matsuda G., Nakajima K., Kawaguchi Y., Yamanashi Y., Hirai K. (2003). Epstein-Barr virus (EBV) nuclear antigen leader protein (EBNA-LP) forms complexes with a cellular anti-apoptosis protein Bcl-2 or its EBV counterpart BHRF1 through HS1-associated protein X-1. Microbiol. Immunol..

[55] Allday M.J., Bazot Q., White R.E. (2015). The EBNA3 Family: Two Oncoproteins and a Tumour Suppressor that Are Central to the Biology of EBV in B Cells. Curr. Top. Microbiol. Immunol..

[56] Le Roux A., Kerdiles B., Walls D., Dedieu J.F., Perricaudet M. (1994). The Epstein-Barr virus determined nuclear antigens EBNA-3A, -3B, and -3C repress EBNA-2-mediated transactivation of the viral terminal protein 1 gene promoter. Virology.

[57] Yenamandra S.P., Sompallae R., Klein G., Kashuba E. (2009). Comparative analysis of the Epstein-Barr virus encoded nuclear proteins of EBNA-3 family. Comput. Biol. Med..

[58] Hertle M.L., Popp C., Petermann S., Maier S., Kremmer E., Lang R., Mages J., Kempkes B. (2009). Differential gene expression patterns of EBV infected EBNA-3A positive and negative human B lymphocytes. PLoS Pathog..

[59] Skalska L., White R.E., Franz M., Ruhmann M., Allday M.J. (2010). Epigenetic repression of p16(INK4A) by latent Epstein-Barr virus requires the interaction of EBNA3A and EBNA3C with CtBP. PLoS Pathog..

[60] Tomkinson B., Robertson E., Kieff E. (1993). Epstein-Barr virus nuclear proteins EBNA-3A and EBNA-3C are essential for B-lymphocyte growth transformation. J. Virol..

[61] Tomkinson B., Kieff E. (1992). Use of second-site homologous recombination to demonstrate that Epstein-Barr virus nuclear protein 3B is not important for lymphocyte infection or growth transformation in vitro. J. Virol..

[62] Chen A., Divisconte M., Jiang X., Quink C., Wang F. (2005). Epstein-Barr virus with the latent infection nuclear antigen 3B completely deleted is still competent for B-cell growth transformation in vitro. J. Virol..

[63] Paschos K., Parker G.A., Watanatanasup E., White R.E., Allday M.J. (2012). BIM promoter directly targeted by EBNA3C in polycomb-mediated repression by EBV. Nucleic Acids Res..

[64] Paschos K., Smith P., Anderton E., Middeldorp J.M., White R.E., Allday M.J. (2009). Epstein-barr virus latency in B cells leads to epigenetic repression and CpG methylation of the tumour suppressor gene Bim. PLoS Pathog..

[65] Anderton E., Yee J., Smith P., Crook T., White R.E., Allday M.J. (2008). Two Epstein-Barr virus (EBV) oncoproteins cooperate to repress expression of the proapoptotic tumour-suppressor Bim: Clues to the pathogenesis of Burkitt’s lymphoma. Oncogene.

[66] Maruo S., Zhao B., Johannsen E., Kieff E., Zou J., Takada K. (2011). Epstein-Barr virus nuclear antigens 3C and 3A maintain lymphoblastoid cell growth by repressing p16INK4A and p14ARF expression. Proc. Natl. Acad. Sci. USA.

[67] Maruo S., Wu Y., Ishikawa S., Kanda T., Iwakiri D., Takada K. (2006). Epstein-Barr virus nuclear protein EBNA3C is required for cell cycle progression and growth maintenance of lymphoblastoid cells. Proc. Natl. Acad. Sci. USA.

[68] Skalska L., White R.E., Parker G.A., Turro E., Sinclair A.J., Paschos K., Allday M.J. (2013). Induction of p16(INK4a) is the major barrier to proliferation when Epstein-Barr virus (EBV) transforms primary B cells into lymphoblastoid cell lines. PLoS Pathog..

[69] Jiang S., Willox B., Zhou H., Holthaus A.M., Wang A., Shi T.T., Maruo S., Kharchenko P.V., Johannsen E.C., Kieff E. (2014). Epstein-Barr virus nuclear antigen 3C binds to BATF/IRF4 or SPI1/IRF4 composite sites and recruits Sin3A to repress CDKN2A. Proc. Natl. Acad. Sci. USA.

[70] Cai Q., Guo Y., Xiao B., Banerjee S., Saha A., Lu J., Glisovic T., Robertson E.S. (2011). Epstein-Barr virus nuclear antigen 3C stabilizes Gemin3 to block p53-mediated apoptosis. PLoS Pathog..

[71] Yi F., Saha A., Murakami M., Kumar P., Knight J.S., Cai Q., Choudhuri T., Robertson E.S. (2009). Epstein-Barr virus nuclear antigen 3C targets p53 and modulates its transcriptional and apoptotic activities. Virology.

[72] Saha A., Murakami M., Kumar P., Bajaj B., Sims K., Robertson E.S. (2009). Epstein-Barr virus nuclear antigen 3C augments Mdm2-mediated p53 ubiquitination and degradation by deubiquitinating Mdm2. J. Virol..

[73] McClellan M.J., Wood C.D., Ojeniyi O., Cooper T.J., Kanhere A., Arvey A., Webb H.M., Palermo R.D., Harth-Hertle M.L., Kempkes B. (2013). Modulation of enhancer looping and differential gene targeting by Epstein-Barr virus transcription factors directs cellular reprogramming. PLoS Pathog..

[74] Sears J., Ujihara M., Wong S., Ott C., Middeldorp J., Aiyar A. (2004). The amino terminus of Epstein-Barr Virus (EBV) nuclear antigen 1 contains AT hooks that facilitate the replication and partitioning of latent EBV genomes by tethering them to cellular chromosomes. J. Virol..

[75] Mackey D., Sugden B. (1999). Applications of oriP plasmids and their mode of replication. Methods Enzymol..

[76] Leight E.R., Sugden B. (2000). EBNA-1: A protein pivotal to latent infection by Epstein-Barr virus. Rev. Med. Virol..

[77] Frappier L. (2012). Contributions of Epstein-Barr nuclear antigen 1 (EBNA1) to cell immortalization and survival. Viruses.

[78] Gruhne B., Sompallae R., Masucci M.G. (2009). Three Epstein-Barr virus latency proteins independently promote genomic instability by inducing DNA damage, inhibiting DNA repair and inactivating cell cycle checkpoints. Oncogene.

[79] Gruhne B., Sompallae R., Marescotti D., Kamranvar S.A., Gastaldello S., Masucci M.G. (2009). The Epstein-Barr virus nuclear antigen-1 promotes genomic instability via induction of reactive oxygen species. Proc. Natl. Acad. Sci. USA.

[80] Saridakis V., Sheng Y., Sarkari F., Holowaty M.N., Shire K., Nguyen T., Zhang R.G., Liao J., Lee W., Edwards A.M. (2005). Structure of the p53 binding domain of HAUSP/USP7 bound to Epstein-Barr nuclear antigen 1 implications for EBV-mediated immortalization. Mol. Cell.

[81] Lu J., Murakami M., Verma S.C., Cai Q.L., Haldar S., Kaul R., Wasik M.A., Middeldorp J., Robertson E.S. (2011). Epstein-Barr Virus nuclear antigen 1 (EBNA1) confers resistance to apoptosis in EBV-positive B-lymphoma cells through up-regulation of survivin. Virology.

[82] Wood V.H., O’Neil J.D., Wei W., Stewart S.E., Dawson C.W., Young L.S. (2007). Epstein-Barr virus-encoded EBNA1 regulates cellular gene transcription and modulates the STAT1 and TGFbeta signaling pathways. Oncogene.

[83] Canaan A., Haviv I., Urban A.E., Schulz V.P., Hartman S., Zhang Z., Palejev D., Deisseroth A.B., Lacy J., Snyder M. (2009). EBNA1 regulates cellular gene expression by binding cellular promoters. Proc. Natl. Acad. Sci. USA.

[84] Dresang L.R., Vereide D.T., Sugden B. (2009). Identifying sites bound by Epstein-Barr virus nuclear antigen 1 (EBNA1) in the human genome: Defining a position-weighted matrix to predict sites bound by EBNA1 in viral genomes. J. Virol..

[85] Lu F., Wikramasinghe P., Norseen J., Tsai K., Wang P., Showe L., Davuluri R.V., Lieberman P.M. (2010). Genome-wide analysis of host-chromosome binding sites for Epstein-Barr Virus Nuclear Antigen 1 (EBNA1). Virol. J..

[86] Kaye K.M., Izumi K.M., Kieff E. (1993). Epstein-Barr virus latent membrane protein 1 is essential for B-lymphocyte growth transformation. Proc. Natl. Acad. Sci. USA.

[87] Dirmeier U., Neuhierl B., Kilger E., Reisbach G., Sandberg M.L., Hammerschmidt W. (2003). Latent membrane protein 1 is critical for efficient growth transformation of human B cells by epstein-barr virus. Cancer Res..

[88] Rastelli J., Homig-Holzel C., Seagal J., Muller W., Hermann A.C., Rajewsky K., Zimber-Strobl U. (2008). LMP1 signaling can replace CD40 signaling in B cells in vivo and has unique features of inducing class-switch recombination to IgG1. Blood.

[89] Uchida J., Yasui T., Takaoka-Shichijo Y., Muraoka M., Kulwichit W., Raab-Traub N., Kikutani H. (1999). Mimicry of CD40 signals by Epstein-Barr virus LMP1 in B lymphocyte responses. Science.

[90] Izumi K.M., Kieff E.D. (1997). The Epstein-Barr virus oncogene product latent membrane protein 1 engages the tumor necrosis factor receptor-associated death domain protein to mediate B lymphocyte growth transformation and activate NF-kappaB. Proc. Natl. Acad. Sci. USA.

[91] Izumi K.M., Kaye K.M., Kieff E.D. (1997). The Epstein-Barr virus LMP1 amino acid sequence that engages tumor necrosis factor receptor associated factors is critical for primary B lymphocyte growth transformation. Proc. Natl. Acad. Sci. USA.

[92] Mosialos G., Birkenbach M., Yalamanchili R., VanArsdale T., Ware C., Kieff E. (1995). The Epstein-Barr virus transforming protein LMP1 engages signaling proteins for the tumor necrosis factor receptor family. Cell.

[93] Luftig M., Prinarakis E., Yasui T., Tsichritzis T., Cahir-McFarland E., Inoue J., Nakano H., Mak T.W., Yeh W.C., Li X. (2003). Epstein-Barr virus latent membrane protein 1 activation of NF-kappaB through IRAK1 and TRAF6. Proc. Natl. Acad. Sci. USA.

[94] Edwards R.H., Marquitz A.R., Raab-Traub N. (2015). Changes in expression induced by Epstein-Barr Virus LMP1-CTAR1: Potential role of bcl3. MBio.

[95] Gewurz B.E., Mar J.C., Padi M., Zhao B., Shinners N.P., Takasaki K., Bedoya E., Zou J.Y., Cahir-McFarland E., Quackenbush J. (2011). Canonical NF-κB activation is essential for Epstein-Barr virus latent membrane protein 1 TES2/CTAR2 gene regulation. J. Virol..

[96] Ersing I., Bernhardt K., Gewurz B.E. (2013). NF-kappaB and IRF7 pathway activation by Epstein-Barr virus Latent Membrane Protein 1. Viruses.

[97] Kung C.P., Raab-Traub N. (2010). Epstein-Barr virus latent membrane protein 1 modulates distinctive NF-κB pathways through C-terminus-activating region 1 to regulate epidermal growth factor receptor expression. J. Virol..

[98] Pratt Z.L., Zhang J., Sugden B. (2012). The latent membrane protein 1 (LMP1) oncogene of Epstein-Barr virus can simultaneously induce and inhibit apoptosis in B cells. J. Virol..

[99] D’Souza B., Rowe M., Walls D. (2000). The bfl-1 gene is transcriptionally upregulated by the Epstein-Barr virus LMP1, and its expression promotes the survival of a Burkitt’s lymphoma cell line. J. Virol..

[100] D’Souza B.N., Edelstein L.C., Pegman P.M., Smith S.M., Loughran S.T., Clarke A., Mehl A., Rowe M., Gelinas C., Walls D. (2004). Nuclear factor κB -dependent activation of the antiapoptotic bfl-1 gene by the Epstein-Barr virus latent membrane protein 1 and activated CD40 receptor. J. Virol..

[101] Henderson S., Rowe M., Gregory C., Croom-Carter D., Wang F., Longnecker R., Kieff E., Rickinson A. (1991). Induction of bcl-2 expression by Epstein-Barr virus latent membrane protein 1 protects infected B cells from programmed cell death. Cell.

[102] Wang S., Rowe M., Lundgren E. (1996). Expression of the Epstein Barr virus transforming protein LMP1 causes a rapid and transient stimulation of the Bcl-2 homologue Mcl-1 levels in B-cell lines. Cancer Res..

[103] Tsai S.C., Lin S.J., Lin C.J., Chou Y.C., Lin J.H., Yeh T.H., Chen M.R., Huang L.M., Lu M.Y., Huang Y.C. (2013). Autocrine CCL3 and CCL4 induced by the oncoprotein LMP1 promote Epstein-Barr virus-triggered B cell proliferation. J. Virol..

[104] Zhao B., Barrera L.A., Ersing I., Willox B., Schmidt S.C., Greenfeld H., Zhou H., Mollo S.B., Shi T.T., Takasaki K. (2014). The NF-κB genomic landscape in lymphoblastoid B cells. Cell Rep..

[105] Ma Y., Walsh M.J., Bernhardt K., Ashbaugh C.W., Trudeau S.J., Ashbaugh I.Y., Jiang S., Jiang C., Zhao B., Root D.E. (2017). CRISPR/Cas9 Screens Reveal Epstein-Barr Virus-Transformed B Cell Host Dependency Factors. Cell Host Microbe.

[106] Le Clorennec C., Ouk T.S., Youlyouz-Marfak I., Panteix S., Martin C.C., Rastelli J., Adriaenssens E., Zimber-Strobl U., Coll J., Feuillard J. (2008). Molecular basis of cytotoxicity of Epstein-Barr virus (EBV) latent membrane protein 1 (LMP1) in EBV latency III B cells: LMP1 induces type II ligand-independent autoactivation of CD95/Fas with caspase 8-mediated apoptosis. J. Virol..

[107] Le Clorennec C., Youlyouz-Marfak I., Adriaenssens E., Coll J., Bornkamm G.W., Feuillard J. (2006). EBV latency III immortalization program sensitizes B cells to induction of CD95-mediated apoptosis via LMP1: Role of NF-κB, STAT1, and p53. Blood.

[108] Laux G., Dugrillon F., Eckert C., Adam B., Zimber-Strobl U., Bornkamm G.W. (1994). Identification and characterization of an Epstein-Barr virus nuclear antigen 2-responsive cis element in the bidirectional promoter region of latent membrane protein and terminal protein 2 genes. J. Virol..

[109] Laux G., Perricaudet M., Farrell P.J. (1988). A spliced Epstein-Barr virus gene expressed in immortalized lymphocytes is created by circularization of the linear viral genome. EMBO J..

[110] Sample J., Liebowitz D., Kieff E. (1989). Two related Epstein-Barr virus membrane proteins are encoded by separate genes. J. Virol..

[111] Kim O.J., Yates J.L. (1993). Mutants of Epstein-Barr virus with a selective marker disrupting the TP gene transform B cells and replicate normally in culture. J. Virol..

[112] Longnecker R., Miller C.L., Tomkinson B., Miao X.Q., Kieff E. (1993). Deletion of DNA encoding the first five transmembrane domains of Epstein-Barr virus latent membrane proteins 2A and 2B. J. Virol..

[113] Longnecker R., Miller C.L., Miao X.Q., Tomkinson B., Kieff E. (1993). The last seven transmembrane and carboxy-terminal cytoplasmic domains of Epstein-Barr virus latent membrane protein 2 (LMP2) are dispensable for lymphocyte infection and growth transformation in vitro. J. Virol..

[114] Speck P., Kline K.A., Cheresh P., Longnecker R. (1999). Epstein-Barr virus lacking latent membrane protein 2 immortalizes B cells with efficiency indistinguishable from that of wild-type virus. J. Gen. Virol..

[115] Fukuda M., Longnecker R. (2007). Epstein-Barr virus latent membrane protein 2A mediates transformation through constitutive activation of the Ras/PI3-K/Akt Pathway. J. Virol..

[116] Cen O., Longnecker R. (2015). Latent Membrane Protein 2 (LMP2). Curr. Top. Microbiol. Immunol..

[117] Mancao C., Altmann M., Jungnickel B., Hammerschmidt W. (2005). Rescue of “crippled” germinal center B cells from apoptosis by Epstein-Barr virus. Blood.

[118] Mancao C., Hammerschmidt W. (2007). Epstein-Barr virus latent membrane protein 2A is a B-cell receptor mimic and essential for B-cell survival. Blood.

[119] Merchant M., Longnecker R. (2001). LMP2A survival and developmental signals are transmitted through Btk-dependent and Btk-independent pathways. Virology.

[120] Merchant M., Swart R., Katzman R.B., Ikeda M., Ikeda A., Longnecker R., Dykstra M.L., Pierce S.K. (2001). The effects of the Epstein-Barr virus latent membrane protein 2A on B cell function. Int. Rev. Immunol..

[121] Swart R., Ruf I.K., Sample J., Longnecker R. (2000). Latent membrane protein 2A-mediated effects on the phosphatidylinositol 3-Kinase/Akt pathway. J. Virol..

[122] Laux G., Economou A., Farrell P.J. (1989). The terminal protein gene 2 of Epstein-Barr virus is transcribed from a bidirectional latent promoter region. J. Gen. Virol..

[123] Rovedo M., Longnecker R. (2008). Epstein-Barr virus latent membrane protein 2A preferentially signals through the Src family kinase Lyn. J. Virol..

[124] Kvansakul M., Hinds M.G. (2013). Structural biology of the Bcl-2 family and its mimicry by viral proteins. Cell Death Dis..

[125] Cooray S., Bahar M.W., Abrescia N.G., McVey C.E., Bartlett N.W., Chen R.A., Stuart D.I., Grimes J.M., Smith G.L. (2007). Functional and structural studies of the vaccinia virus virulence factor N1 reveal a Bcl-2-like anti-apoptotic protein. J. Gen. Virol..

[126] Aoyagi M., Zhai D., Jin C., Aleshin A.E., Stec B., Reed J.C., Liddington R.C. (2007). Vaccinia virus N1L protein resembles a B cell lymphoma-2 (Bcl-2) family protein. Protein Sci..

[127] DiPerna G., Stack J., Bowie A.G., Boyd A., Kotwal G., Zhang Z., Arvikar S., Latz E., Fitzgerald K.A., Marshall W.L. (2004). Poxvirus protein N1L targets the I-κB kinase complex, inhibits signaling to NF-κB by the tumor necrosis factor superfamily of receptors, and inhibits NF-κB and IRF3 signaling by toll-like receptors. J. Biol. Chem..

[128] Graham S.C., Bahar M.W., Cooray S., Chen R.A., Whalen D.M., Abrescia N.G., Alderton D., Owens R.J., Stuart D.I., Smith G.L. (2008). Vaccinia virus proteins A52 and B14 Share a Bcl-2-like fold but have evolved to inhibit NF-κB rather than apoptosis. PLoS Pathog..

[129] Altmann M., Hammerschmidt W. (2005). Epstein-Barr virus provides a new paradigm: A requirement for the immediate inhibition of apoptosis. PLoS Biol..

[130] Marshall W.L., Yim C., Gustafson E., Graf T., Sage D.R., Hanify K., Williams L., Fingeroth J., Finberg R.W. (1999). Epstein-Barr virus encodes a novel homolog of the bcl-2 oncogene that inhibits apoptosis and associates with Bax and Bak. J. Virol..

[131] Henderson S., Huen D., Rowe M., Dawson C., Johnson G., Rickinson A. (1993). Epstein-Barr virus-coded BHRF1 protein, a viral homologue of Bcl-2, protects human B cells from programmed cell death. Proc. Natl. Acad. Sci. USA.

[132] Foight G.W., Keating A.E. (2015). Locating Herpesvirus Bcl-2 Homologs in the Specificity Landscape of Anti-Apoptotic Bcl-2 Proteins. J. Mol. Biol..

[133] Kvansakul M., Wei A.H., Fletcher J.I., Willis S.N., Chen L., Roberts A.W., Huang D.C., Colman P.M. (2010). Structural basis for apoptosis inhibition by Epstein-Barr virus BHRF1. PLoS Pathog..

[134] Flanagan A.M., Letai A. (2008). BH3 domains define selective inhibitory interactions with BHRF-1 and KSHV BCL-2. Cell Death Differ..

[135] Desbien A.L., Kappler J.W., Marrack P. (2009). The Epstein-Barr virus Bcl-2 homolog, BHRF1, blocks apoptosis by binding to a limited amount of Bim. Proc. Natl. Acad. Sci. USA.

[136] Fanidi A., Hancock D.C., Littlewood T.D. (1998). Suppression of c-Myc-induced apoptosis by the Epstein-Barr virus gene product BHRF1. J. Virol..

[137] Foghsgaard L., Jaattela M. (1997). The ability of BHRF1 to inhibit apoptosis is dependent on stimulus and cell type. J. Virol..

[138] Kawanishi M., Tada-Oikawa S., Kawanishi S. (2002). Epstein-Barr virus BHRF1 functions downstream of Bid cleavage and upstream of mitochondrial dysfunction to inhibit TRAIL-induced apoptosis in BJAB cells. Biochem. Biophys. Res. Commun..

[139] McCarthy N.J., Hazlewood S.A., Huen D.S., Rickinson A.B., Williams G.T. (1996). The Epstein-Barr virus gene BHRF1, a homologue of the cellular oncogene Bcl-2, inhibits apoptosis induced by gamma radiation and chemotherapeutic drugs. Adv. Exp. Med. Biol..

[140] Watanabe A., Maruo S., Ito T., Ito M., Katsumura K.R., Takada K. (2010). Epstein-Barr virus-encoded Bcl-2 homologue functions as a survival factor in Wp-restricted Burkitt lymphoma cell line P3HR-1. J. Virol..

[141] Yee J., White R.E., Anderton E., Allday M.J. (2011). Latent Epstein-Barr Virus Can Inhibit Apoptosis in B Cells by Blocking the Induction of NOXA Expression. PLoS ONE.

[142] Zhao E.G., Song Q., Cross S., Misko I., Lees-Miller S.P., Lavin M.F. (1998). Resistance to etoposide-induced apoptosis in a Burkitt’s lymphoma cell line. Int. J. Cancer.

[143] Bellows D.S., Howell M., Pearson C., Hazlewood S.A., Hardwick J.M. (2002). Epstein-Barr virus BALF1 is a BCL-2-like antagonist of the herpesvirus antiapoptotic BCL-2 proteins. J. Virol..

[144] Chen L., Willis S.N., Wei A., Smith B.J., Fletcher J.I., Hinds M.G., Colman P.M., Day C.L., Adams J.M., Huang D.C. (2005). Differential targeting of prosurvival Bcl-2 proteins by their BH3-only ligands allows complementary apoptotic function. Mol. Cell.

[145] Willis S.N., Chen L., Dewson G., Wei A., Naik E., Fletcher J.I., Adams J.M., Huang D.C. (2005). Proapoptotic Bak is sequestered by Mcl-1 and Bcl-xL, but not Bcl-2, until displaced by BH3-only proteins. Genes. Dev..

[146] Fletcher J.I., Meusburger S., Hawkins C.J., Riglar D.T., Lee E.F., Fairlie W.D., Huang D.C., Adams J.M. (2008). Apoptosis is triggered when prosurvival Bcl-2 proteins cannot restrain Bax. Proc. Natl. Acad. Sci. USA.

[147] Smits C., Czabotar P.E., Hinds M.G., Day C.L. (2008). Structural plasticity underpins promiscuous binding of the prosurvival protein A1. Structure.

[148] Pettersen E.F., Goddard T.D., Huang C.C., Couch G.S., Greenblatt D.M., Meng E.C., Ferrin T.E. (2004). UCSF Chimera—A visualization system for exploratory research and analysis. J. Comput. Chem..

[149] Lerner M.R., Andrews N.C., Miller G., Steitz J.A. (1981). Two small RNAs encoded by Epstein-Barr virus and complexed with protein are precipitated by antibodies from patients with systemic lupus erythematosus. Proc. Natl. Acad. Sci. USA.

[150] Shannon-Lowe C., Adland E., Bell A.I., Delecluse H.J., Rickinson A.B., Rowe M. (2009). Features distinguishing Epstein-Barr virus infections of epithelial cells and B cells: Viral genome expression, genome maintenance, and genome amplification. J. Virol..

[151] Zhu J.Y., Pfuhl T., Motsch N., Barth S., Nicholls J., Grasser F., Meister G. (2009). Identification of novel Epstein-Barr virus microRNA genes from nasopharyngeal carcinomas. J. Virol..

[152] Chen S.J., Chen G.H., Chen Y.H., Liu C.Y., Chang K.P., Chang Y.S., Chen H.C. (2010). Characterization of Epstein-Barr virus miRNAome in nasopharyngeal carcinoma by deep sequencing. PLoS ONE.

[153] Grundhoff A., Sullivan C.S., Ganem D. (2006). A combined computational and microarray-based approach identifies novel microRNAs encoded by human gamma-herpesviruses. RNA.

[154] Edwards R.H., Marquitz A.R., Raab-Traub N. (2008). Epstein-Barr virus BART microRNAs are produced from a large intron prior to splicing. J. Virol..

[155] Bartel D.P. (2004). MicroRNAs: Genomics, biogenesis, mechanism, and function. Cell.

[156] Ambros V. (2004). The functions of animal microRNAs. Nature.

[157] Swaminathan S., Tomkinson B., Kieff E. (1991). Recombinant Epstein-Barr virus with small RNA (EBER) genes deleted transforms lymphocytes and replicates in vitro. Proc. Natl. Acad. Sci. USA.

[158] Swaminathan S., Huneycutt B.S., Reiss C.S., Kieff E. (1992). Epstein-Barr virus-encoded small RNAs (EBERs) do not modulate interferon effects in infected lymphocytes. J. Virol..

[159] Yajima M., Kanda T., Takada K. (2005). Critical role of Epstein-Barr Virus (EBV)-encoded RNA in efficient EBV-induced B-lymphocyte growth transformation. J. Virol..

[160] Wu Y., Maruo S., Yajima M., Kanda T., Takada K. (2007). Epstein-Barr virus (EBV)-encoded RNA 2 (EBER2) but not EBER1 plays a critical role in EBV-induced B-cell growth transformation. J. Virol..

[161] Gregorovic G., Bosshard R., Karstegl C.E., White R.E., Pattle S., Chiang A.K., Dittrich-Breiholz O., Kracht M., Russ R., Farrell P.J. (2011). Cellular gene expression that correlates with EBER expression in Epstein-Barr Virus-infected lymphoblastoid cell lines. J. Virol..

[162] Benetti R., Del Sal G., Monte M., Paroni G., Brancolini C., Schneider C. (2001). The death substrate Gas2 binds m-calpain and increases susceptibility to p53-dependent apoptosis. EMBO J..

[163] Burgess J.T., Bolderson E., Adams M.N., Baird A.M., Zhang S.D., Gately K.A., Umezawa K., O’Byrne K.J., Richard D.J. (2016). Activation and cleavage of SASH1 by caspase-3 mediates an apoptotic response. Cell Death Dis..

[164] Xia T., O’Hara A., Araujo I., Barreto J., Carvalho E., Sapucaia J.B., Ramos J.C., Luz E., Pedroso C., Manrique M. (2008). EBV microRNAs in primary lymphomas and targeting of CXCL-11 by ebv-mir-BHRF1–3. Cancer Res..

[165] Skalsky R.L., Corcoran D.L., Gottwein E., Frank C.L., Kang D., Hafner M., Nusbaum J.D., Feederle R., Delecluse H.J., Luftig M.A. (2012). The viral and cellular microRNA targetome in lymphoblastoid cell lines. PLoS Pathog..

[166] Pratt Z.L., Kuzembayeva M., Sengupta S., Sugden B. (2009). The microRNAs of Epstein-Barr Virus are expressed at dramatically differing levels among cell lines. Virology.

[167] Feederle R., Haar J., Bernhardt K., Linnstaedt S.D., Bannert H., Lips H., Cullen B.R., Delecluse H.J. (2011). The members of an Epstein-Barr virus microRNA cluster cooperate to transform B lymphocytes. J. Virol..

[168] Feederle R., Linnstaedt S.D., Bannert H., Lips H., Bencun M., Cullen B.R., Delecluse H.J. (2011). A viral microRNA cluster strongly potentiates the transforming properties of a human herpesvirus. PLoS Pathog..

[169] Seto E., Moosmann A., Gromminger S., Walz N., Grundhoff A., Hammerschmidt W. (2010). Micro RNAs of Epstein-Barr virus promote cell cycle progression and prevent apoptosis of primary human B cells. PLoS Pathog..

[170] Wahl A., Linnstaedt S.D., Esoda C., Krisko J.F., Martinez-Torres F., Delecluse H.J., Cullen B.R., Garcia J.V. (2013). A cluster of virus-encoded microRNAs accelerates acute systemic Epstein-Barr virus infection but does not significantly enhance virus-induced oncogenesis in vivo. J. Virol..

[171] Majoros W.H., Lekprasert P., Mukherjee N., Skalsky R.L., Corcoran D.L., Cullen B.R., Ohler U. (2013). MicroRNA target site identification by integrating sequence and binding information. Nat. Methods.

[172] Skalsky R.L., Cullen B.R. (2015). EBV Noncoding RNAs. Curr. Top. Microbiol. Immunol..

[173] Song M.S., Salmena L., Pandolfi P.P. (2012). The functions and regulation of the PTEN tumour suppressor. Nat. Rev. Mol. Cell Biol..

[174] Warnatz H.J., Schmidt D., Manke T., Piccini I., Sultan M., Borodina T., Balzereit D., Wruck W., Soldatov A., Vingron M. (2011). The BTB and CNC homology 1 (BACH1) target genes are involved in the oxidative stress response and in control of the cell cycle. J. Biol. Chem..

[175] Castellini L., Moon E.J., Razorenova O.V., Krieg A.J., von Eyben R., Giaccia A.J. (2017). KDM4B/JMJD2B is a p53 target gene that modulates the amplitude of p53 response after DNA damage. Nucleic Acids Res..

[176] Gilligan K.J., Rajadurai P., Lin J.C., Busson P., Abdel-Hamid M., Prasad U., Tursz T., Raab-Traub N. (1991). Expression of the Epstein-Barr virus BamHI A fragment in nasopharyngeal carcinoma: Evidence for a viral protein expressed in vivo. J. Virol..

[177] Hitt M.M., Allday M.J., Hara T., Karran L., Jones M.D., Busson P., Tursz T., Ernberg I., Griffin B.E. (1989). EBV gene expression in an NPC-related tumour. EMBO J..

[178] Sadler R.H., Raab-Traub N. (1995). Structural analyses of the Epstein-Barr virus BamHI A transcripts. J. Virol..

[179] Smith P.R., Gao Y., Karran L., Jones M.D., Snudden D., Griffin B.E. (1993). Complex nature of the major viral polyadenylated transcripts in Epstein-Barr virus-associated tumors. J. Virol..

[180] Zhang J., Chen H., Weinmaster G., Hayward S.D. (2001). Epstein-Barr virus BamHi-a rightward transcript-encoded RPMS protein interacts with the CBF1-associated corepressor CIR to negatively regulate the activity of EBNA2 and NotchIC. J. Virol..

[181] Smith P.R., de Jesus O., Turner D., Hollyoake M., Karstegl C.E., Griffin B.E., Karran L., Wang Y., Hayward S.D., Farrell P.J. (2000). Structure and coding content of CST (BART) family RNAs of Epstein-Barr virus. J. Virol..

[182] Kusano S., Raab-Traub N. (2001). An Epstein-Barr virus protein interacts with Notch. J. Virol..

[183] Al-Mozaini M., Bodelon G., Karstegl C.E., Jin B., Al-Ahdal M., Farrell P.J. (2009). Epstein-Barr virus BART gene expression. J. Gen. Virol..

[184] Van Beek J., Brink A.A., Vervoort M.B., van Zijp M.J., Meijer C.J., van den Brule A.J., Middeldorp J.M. (2003). In vivo transcription of the Epstein-Barr virus (EBV) BamHI-A region without associated in vivo BARF0 protein expression in multiple EBV-associated disorders. J. Gen. Virol..

[185] Bornkamm G.W., Delius H., Zimber U., Hudewentz J., Epstein M.A. (1980). Comparison of Epstein-Barr virus strains of different origin by analysis of the viral DNAs. J. Virol..

[186] Raab-Traub N., Dambaugh T., Kieff E. (1980). DNA of Epstein-Barr virus VIII: B95–8, the previous prototype, is an unusual deletion derivative. Cell.

[187] Vereide D.T., Seto E., Chiu Y.F., Hayes M., Tagawa T., Grundhoff A., Hammerschmidt W., Sugden B. (2014). Epstein-Barr virus maintains lymphomas via its miRNAs. Oncogene.

[188] Dolken L., Malterer G., Erhard F., Kothe S., Friedel C.C., Suffert G., Marcinowski L., Motsch N., Barth S., Beitzinger M. (2010). Systematic analysis of viral and cellular microRNA targets in cells latently infected with human gamma-herpesviruses by RISC immunoprecipitation assay. Cell Host Microbe.

[189] Kang H.S., Ock J., Lee H.J., Lee Y.J., Kwon B.M., Hong S.H. (2013). Early growth response protein 1 upregulation and nuclear translocation by 2’-benzoyloxycinnamaldehyde induces prostate cancer cell death. Cancer Lett..

[190] Bellot G., Cartron P.F., Er E., Oliver L., Juin P., Armstrong L.C., Bornstein P., Mihara K., Manon S., Vallette F.M. (2007). TOM22, a core component of the mitochondria outer membrane protein translocation pore, is a mitochondrial receptor for the proapoptotic protein Bax. Cell Death Differ..

[191] Chi S.W., Zang J.B., Mele A., Darnell R.B. (2009). Argonaute HITS-CLIP decodes microRNA-mRNA interaction maps. Nature.

[192] Riley K.J., Rabinowitz G.S., Yario T.A., Luna J.M., Darnell R.B., Steitz J.A. (2012). EBV and human microRNAs co-target oncogenic and apoptotic viral and human genes during latency. EMBO J..

[193] Lin Z., Xu G., Deng N., Taylor C., Zhu D., Flemington E.K. (2010). Quantitative and qualitative RNA-Seq-based evaluation of Epstein-Barr virus transcription in type I latency Burkitt’s lymphoma cells. J. Virol..

[194] Moss W.N., Steitz J.A. (2013). Genome-wide analyses of Epstein-Barr virus reveal conserved RNA structures and a novel stable intronic sequence RNA. BMC Genom..

[195] Thorley-Lawson D.A. (2015). EBV Persistence—Introducing the Virus. Curr. Top. Microbiol. Immunol..

[196] Vrzalikova K., Vockerodt M., Leonard S., Bell A., Wei W., Schrader A., Wright K.L., Kube D., Rowe M., Woodman C.B. (2011). Down-regulation of BLIMP1alpha by the EBV oncogene, LMP-1, disrupts the plasma cell differentiation program and prevents viral replication in B cells: Implications for the pathogenesis of EBV-associated B-cell lymphomas. Blood.

[197] Sinclair A.J. (2013). Epigenetic control of Epstein-Barr virus transcription—Relevance to viral life cycle?. Front. Genet..

[198] Niller H.H., Wolf H., Minarovits J. (2009). Epigenetic dysregulation of the host cell genome in Epstein-Barr virus-associated neoplasia. Semin. Cancer Biol..

[199] Kalla M., Schmeinck A., Bergbauer M., Pich D., Hammerschmidt W. (2010). AP-1 homolog BZLF1 of Epstein-Barr virus has two essential functions dependent on the epigenetic state of the viral genome. Proc. Natl. Acad. Sci. USA.

[200] Woellmer A., Arteaga-Salas J.M., Hammerschmidt W. (2012). BZLF1 governs CpG-methylated chromatin of Epstein-Barr Virus reversing epigenetic repression. PLoS Pathog..

[201] Gruffat H., Sergeant A. (1994). Characterization of the DNA-binding site repertoire for the Epstein-Barr virus transcription factor R. Nucleic Acids Res..

[202] Gutsch D.E., Marcu K.B., Kenney S.C. (1994). The Epstein-Barr virus BRLF1 gene product transactivates the murine and human c-myc promoters. Cell. Mol. Biol..

[203] Ragoczy T., Miller G. (2001). Autostimulation of the Epstein-Barr virus BRLF1 promoter is mediated through consensus Sp1 and Sp3 binding sites. J. Virol..

[204] Hammerschmidt W., Sugden B. (1988). Identification and characterization of oriLyt, a lytic origin of DNA replication of Epstein-Barr virus. Cell.

[205] Kawanishi M. (1993). Epstein-Barr virus induces fragmentation of chromosomal DNA during lytic infection. J. Virol..

[206] Morrison T.E., Kenney S.C. (2004). BZLF1, an Epstein-Barr virus immediate-early protein, induces p65 nuclear translocation while inhibiting p65 transcriptional function. Virology.

[207] Zuo J., Thomas W.A., Haigh T.A., Fitzsimmons L., Long H.M., Hislop A.D., Taylor G.S., Rowe M. (2011). Epstein-Barr virus evades CD4+ T cell responses in lytic cycle through BZLF1-mediated downregulation of CD74 and the cooperation of vBcl-2. PLoS Pathog..

[208] Williams L.R., Quinn L.L., Rowe M., Zuo J. (2015). Induction of the Lytic Cycle Sensitizes Epstein-Barr Virus-Infected B Cells to NK Cell Killing That Is Counteracted by Virus-Mediated NK Cell Evasion Mechanisms in the Late Lytic Cycle. J. Virol..

[209] Inman G.J., Binne U.K., Parker G.A., Farrell P.J., Allday M.J. (2001). Activators of the Epstein-Barr Virus Lytic Program Concomitantly Induce Apoptosis, but Lytic Gene Expression Protects from Cell Death. J. Virol..

[210] Oussaief L., Hippocrate A., Clybouw C., Rampanou A., Ramirez V., Desgranges C., Vazquez A., Khelifa R., Joab I. (2009). Activation of the lytic program of the Epstein-Barr virus in Burkitt’s lymphoma cells leads to a two steps downregulation of expression of the proapoptotic protein BimEL, one of which is EBV-late-gene expression dependent. Virology.

[211] Wen W., Iwakiri D., Yamamoto K., Maruo S., Kanda T., Takada K. (2007). Epstein-Barr virus BZLF1 gene, a switch from latency to lytic infection, is expressed as an immediate-early gene after primary infection of B lymphocytes. J. Virol..

[212] Zeidler R., Eissner G., Meissner P., Uebel S., Tampe R., Lazis S., Hammerschmidt W. (1997). Downregulation of TAP1 in B lymphocytes by cellular and Epstein-Barr virus-encoded interleukin-10. Blood.

[213] Kim H., Choi H., Lee S.K. (2015). Epstein-Barr Virus MicroRNA miR-BART20–5p Suppresses Lytic Induction by Inhibiting BAD-Mediated caspase-3-Dependent Apoptosis. J. Virol..

[214] Yuan J., Cahir-McFarland E., Zhao B., Kieff E. (2006). Virus and cell RNAs expressed during Epstein-Barr virus replication. J. Virol..

[215] Nikitin P.A., Price A.M., McFadden K., Yan C.M., Luftig M.A. (2014). Mitogen-induced B-cell proliferation activates Chk2-dependent G1/S cell cycle arrest. PLoS ONE.

[216] Nikitin P.A., Yan C.M., Forte E., Bocedi A., Tourigny J.P., White R.E., Allday M.J., Patel A., Dave S.S., Kim W. (2010). An ATM/Chk2-mediated DNA damage-responsive signaling pathway suppresses Epstein-Barr virus transformation of primary human B cells. Cell Host Microbe.

[217] McFadden K., Hafez A.Y., Kishton R., Messinger J.E., Nikitin P.A., Rathmell J.C., Luftig M.A. (2016). Metabolic stress is a barrier to Epstein-Barr virus-mediated B-cell immortalization. Proc. Natl. Acad. Sci. USA.

[218] Allday M.J., Sinclair A., Parker G., Crawford D.H., Farrell P.J. (1995). Epstein-Barr virus efficiently immortalizes human B cells without neutralizing the function of p53. EMBO J..

[219] Bernasconi M., Ueda S., Krukowski P., Bornhauser B.C., Ladell K., Dorner M., Sigrist J.A., Campidelli C., Aslandogmus R., Alessi D. (2013). Early gene expression changes by Epstein-Barr virus infection of B-cells indicate CDKs and survivin as therapeutic targets for post-transplant lymphoproliferative diseases. Int. J. Cancer.

[220] Szekely L., Pokrovskaja K., Jiang W.Q., Selivanova G., Lowbeer M., Ringertz N., Wiman K.G., Klein G. (1995). Resting B-cells, EBV-infected B-blasts and established lymphoblastoid cell lines differ in their Rb, p53 and EBNA-5 expression patterns. Oncogene.

[221] Levine A.J. (2009). The common mechanisms of transformation by the small DNA tumor viruses: The inactivation of tumor suppressor gene products: P53. Virology.

[222] Forte E., Luftig M.A. (2009). MDM2-dependent inhibition of p53 is required for Epstein-Barr virus B-cell growth transformation and infected-cell survival. J. Virol..

[223] Shumilov A., Tsai M.H., Schlosser Y.T., Kratz A.S., Bernhardt K., Fink S., Mizani T., Lin X., Jauch A., Mautner J. (2017). Epstein-Barr virus particles induce centrosome amplification and chromosomal instability. Nat. Commun..

[224] Jha H.C., Yang K., El-Naccache D.W., Sun Z., Robertson E.S. (2015). EBNA3C regulates p53 through induction of Aurora kinase B. Oncotarget.

[225] Saha A., Bamidele A., Murakami M., Robertson E.S. (2011). EBNA3C attenuates the function of p53 through interaction with inhibitor of growth family proteins 4 and 5. J. Virol..

[226] Kashuba E., Yurchenko M., Yenamandra S.P., Snopok B., Szekely L., Bercovich B., Ciechanover A., Klein G. (2011). Epstein-Barr virus-encoded EBNA-5 forms trimolecular protein complexes with MDM2 and p53 and inhibits the transactivating function of p53. Int. J. Cancer.

[227] Strasser A., Cory S., Adams J.M. (2011). Deciphering the rules of programmed cell death to improve therapy of cancer and other diseases. EMBO J..

[228] Kelly G.L., Strasser A. (2011). The essential role of evasion from cell death in cancer. Adv. Cancer Res..

[229] Gregory C.D., Dive C., Henderson S., Smith C.A., Williams G.T., Gordon J., Rickinson A.B. (1991). Activation of Epstein-Barr virus latent genes protects human B cells from death by apoptosis. Nature.

[230] Henderson E., Miller G., Robinson J., Heston L. (1977). Efficiency of transformation of lymphocytes by Epstein-Barr virus. Virology.

[231] Sugden B., Mark W. (1977). Clonal transformation of adult human leukocytes by Epstein-Barr virus. J. Virol..

[232] Kanda T., Furuse Y., Oshitani H., Kiyono T. (2016). Highly Efficient CRISPR/Cas9-Mediated Cloning and Functional Characterization of Gastric Cancer-Derived Epstein-Barr Virus Strains. J. Virol..

[233] Harris-Arnold A., Arnold C.P., Schaffert S., Hatton O., Krams S.M., Esquivel C.O., Martinez O.M. (2015). Epstein-Barr virus modulates host cell microRNA-194 to promote IL-10 production and B lymphoma cell survival. Am. J. Transplant..

[234] Hatton O., Lambert S.L., Phillips L.K., Vaysberg M., Natkunam Y., Esquivel C.O., Krams S.M., Martinez O.M. (2013). Syk-induced phosphatidylinositol-3-kinase activation in Epstein-Barr virus posttransplant lymphoproliferative disorder. Am. J. Transplant..

[235] Ghigna M.R., Reineke T., Rince P., Schuffler P., El Mchichi B., Fabre M., Jacquemin E., Durrbach A., Samuel D., Joab I. (2013). Epstein-Barr virus infection and altered control of apoptotic pathways in posttransplant lymphoproliferative disorders. Pathobiology.

[236] Magrath I. (2012). Epidemiology: Clues to the pathogenesis of Burkitt lymphoma. Br. J. Haematol..

[237] Levine P.H., Kamaraju L.S., Connelly R.R., Berard C.W., Dorfman R.F., Magrath I., Easton J.M. (1982). The American Burkitt’s Lymphoma Registry: Eight years’ experience. Cancer.

[238] Araujo I., Foss H.D., Bittencourt A., Hummel M., Demel G., Mendonca N., Herbst H., Stein H. (1996). Expression of Epstein-Barr virus-gene products in Burkitt’s lymphoma in Northeast Brazil. Blood.

[239] Queiroga E.M., Gualco G., Weiss L.M., Dittmer D.P., Araujo I., Klumb C.E., Harrington W.J., Bacchi C.E. (2008). Burkitt lymphoma in Brazil is characterized by geographically distinct clinicopathologic features. Am. J. Clin. Pathol..

[240] Manolov G., Manolova Y. (1972). Marker band in one chromosome 14 from Burkitt lymphomas. Nature.

[241] Zech L., Haglund U., Nilsson K., Klein G. (1976). Characteristic chromosomal abnormalities in biopsies and lymphoid-cell lines from patients with Burkitt and non-Burkitt lymphomas. Int. J. Cancer.

[242] Dalla-Favera R., Bregni M., Erikson J., Patterson D., Gallo R.C., Croce C.M. (1982). Human c-myc onc gene is located on the region of chromosome 8 that is translocated in Burkitt lymphoma cells. Proc. Natl. Acad. Sci. USA.

[243] Adams J.M., Gerondakis S., Webb E., Corcoran L.M., Cory S. (1983). Cellular myc oncogene is altered by chromosome translocation to an immunoglobulin locus in murine plasmacytomas and is rearranged similarly in human Burkitt lymphomas. Proc. Natl. Acad. Sci. USA.

[244] Adams J.M., Harris A.W., Pinkert C.A., Corcoran L.M., Alexander W.S., Cory S., Palmiter R.D., Brinster R.L. (1985). The c-myc oncogene driven by immunoglobulin enhancers induces lymphoid malignancy in transgenic mice. Nature.

[245] Schmidt E.V. (1999). The role of c-myc in cellular growth control. Oncogene.

[246] Pelengaris S., Khan M., Evan G. (2002). c-MYC: More than just a matter of life and death. Nat. Rev. Cancer.

[247] Gaidano G., Ballerini P., Gong J.Z., Inghirami G., Neri A., Newcomb E.W., Magrath I.T., Knowles D.M., Dalla-Favera R. (1991). p53 mutations in human lymphoid malignancies: Association with Burkitt lymphoma and chronic lymphocytic leukemia. Proc. Natl. Acad. Sci. USA.

[248] Farrell P.J., Allan G.J., Shanahan F., Vousden K.H., Crook T. (1991). p53 is frequently mutated in Burkitt’s lymphoma cell lines. EMBO J..

[249] Vousden K.H., Crook T., Farrell P.J. (1993). Biological activities of p53 mutants in Burkitt’s lymphoma cells. J. Gen. Virol..

[250] Cherney B.W., Bhatia K.G., Sgadari C., Gutierrez M.I., Mostowski H., Pike S.E., Gupta G., Magrath I.T., Tosato G. (1997). Role of the p53 tumor suppressor gene in the tumorigenicity of Burkitt’s lymphoma cells. Cancer Res..

[251] Eischen C.M., Weber J.D., Roussel M.F., Sherr C.J., Cleveland J.L. (1999). Disruption of the ARF-Mdm2-p53 tumor suppressor pathway in Myc-induced lymphomagenesis. Genes Dev..

[252] Lindstrom M.S., Klangby U., Wiman K.G. (2001). p14ARF homozygous deletion or MDM2 overexpression in Burkitt lymphoma lines carrying wild type p53. Oncogene.

[253] Egle A., Harris A.W., Bouillet P., Cory S. (2004). Bim is a suppressor of Myc-induced mouse B cell leukemia. Proc. Natl. Acad. Sci. USA.

[254] Eischen C.M., Woo D., Roussel M.F., Cleveland J.L. (2001). Apoptosis triggered by Myc-induced suppression of Bcl-X(L) or Bcl-2 is bypassed during lymphomagenesis. Mol. Cell. Biol..

[255] Maclean K.H., Keller U.B., Rodriguez-Galindo C., Nilsson J.A., Cleveland J.L. (2003). c-Myc augments gamma irradiation-induced apoptosis by suppressing Bcl-XL. Mol. Cell. Biol..

[256] Juin P., Hunt A., Littlewood T., Griffiths B., Swigart L.B., Korsmeyer S., Evan G. (2002). c-Myc functionally cooperates with Bax to induce apoptosis. Mol. Cell. Biol..

[257] Mitchell K.O., Ricci M.S., Miyashita T., Dicker D.T., Jin Z., Reed J.C., El-Deiry W.S. (2000). Bax is a transcriptional target and mediator of c-myc-induced apoptosis. Cancer Res..

[258] Michalak E.M., Jansen E.S., Happo L., Cragg M.S., Tai L., Smyth G.K., Strasser A., Adams J.M., Scott C.L. (2009). Puma and to a lesser extent Noxa are suppressors of Myc-induced lymphomagenesis. Cell Death Differ..

[259] Happo L., Cragg M.S., Phipson B., Haga J.M., Jansen E.S., Herold M.J., Dewson G., Michalak E.M., Vandenberg C.J., Smyth G.K. (2010). Maximal killing of lymphoma cells by DNA damage-inducing therapy requires not only the p53 targets Puma and Noxa, but also Bim. Blood.

[260] Garrison S.P., Jeffers J.R., Yang C., Nilsson J.A., Hall M.A., Rehg J.E., Yue W., Yu J., Zhang L., Onciu M. (2008). Selection against PUMA gene expression in Myc-driven B-cell lymphomagenesis. Mol. Cell. Biol..

[261] Piazza R., Magistroni V., Mogavero A., Andreoni F., Ambrogio C., Chiarle R., Mologni L., Bachmann P.S., Lock R.B., Collini P. (2013). Epigenetic silencing of the proapoptotic gene BIM in anaplastic large cell lymphoma through an MeCP2/SIN3a deacetylating complex. Neoplasia.

[262] Abate F., Ambrosio M.R., Mundo L., Laginestra M.A., Fuligni F., Rossi M., Zairis S., Gazaneo S., De Falco G., Lazzi S. (2015). Distinct Viral and Mutational Spectrum of Endemic Burkitt Lymphoma. PLoS Pathog..

[263] Adhikary S., Eilers M. (2005). Transcriptional regulation and transformation by Myc proteins. Nat. Rev. Mol. Cell Biol..

[264] Dang C.V., O’Donnell K.A., Juopperi T. (2005). The great MYC escape in tumorigenesis. Cancer Cell.

[265] Love C., Sun Z., Jima D., Li G., Zhang J., Miles R., Richards K.L., Dunphy C.H., Choi W.W., Srivastava G. (2012). The genetic landscape of mutations in Burkitt lymphoma. Nat. Genet..

[266] Hemann M.T., Bric A., Teruya-Feldstein J., Herbst A., Nilsson J.A., Cordon-Cardo C., Cleveland J.L., Tansey W.P., Lowe S.W. (2005). Evasion of the p53 tumour surveillance network by tumour-derived MYC mutants. Nature.

[267] Kaymaz Y., Oduor C.I., Yu H., Otieno J.A., Ong’echa J.M., Moormann A.M., Bailey J.A. (2017). Comprehensive Transcriptome and Mutational Profiling of Endemic Burkitt Lymphoma Reveals EBV Type-Specific Differences. Mol. Cancer Res..

[268] Schmitz R., Young R.M., Ceribelli M., Jhavar S., Xiao W., Zhang M., Wright G., Shaffer A.L., Hodson D.J., Buras E. (2012). Burkitt lymphoma pathogenesis and therapeutic targets from structural and functional genomics. Nature.

[269] Richter J., Schlesner M., Hoffmann S., Kreuz M., Leich E., Burkhardt B., Rosolowski M., Ammerpohl O., Wagener R., Bernhart S.H. (2012). Recurrent mutation of the ID3 gene in Burkitt lymphoma identified by integrated genome, exome and transcriptome sequencing. Nat. Genet..

[270] Rowe M., Fitzsimmons L., Bell A.I. (2014). Epstein-Barr virus and Burkitt lymphoma. Chin. J. Cancer.

[271] Kennedy G., Komano J., Sugden B. (2003). Epstein-Barr virus provides a survival factor to Burkitt’s lymphomas. Proc. Natl. Acad. Sci. USA.

[272] Nasimuzzaman M., Kuroda M., Dohno S., Yamamoto T., Iwatsuki K., Matsuzaki S., Mohammad R., Kumita W., Mizuguchi H., Hayakawa T. (2005). Eradication of Epstein-Barr virus episome and associated inhibition of infected tumor cell growth by adenovirus vector-mediated transduction of dominant-negative EBNA1. Mol. Ther..

[273] Amato T., Abate F., Piccaluga P., Iacono M., Fallerini C., Renieri A., De Falco G., Ambrosio M.R., Mourmouras V., Ogwang M. (2016). Clonality Analysis of Immunoglobulin Gene Rearrangement by Next-Generation Sequencing in Endemic Burkitt Lymphoma Suggests Antigen Drive Activation of BCR as Opposed to Sporadic Burkitt Lymphoma. Am. J. Clin. Pathol..

[274] Bellan C., Lazzi S., Hummel M., Palummo N., de Santi M., Amato T., Nyagol J., Sabattini E., Lazure T., Pileri S.A. (2005). Immunoglobulin gene analysis reveals 2 distinct cells of origin for EBV-positive and EBV-negative Burkitt lymphomas. Blood.

[275] Capello D., Scandurra M., Poretti G., Rancoita P.M., Mian M., Gloghini A., Deambrogi C., Martini M., Rossi D., Greiner T.C. (2010). Genome wide DNA-profiling of HIV-related B-cell lymphomas. Br. J. Haematol..

[276] Shiramizu B., McGrath M.S. (1991). Molecular pathogenesis of AIDS-associated non-Hodgkin’s lymphoma. Hematol. Oncol. Clin. N. Am..

[277] Pelicci P.G., Knowles D.M., Magrath I., Dalla-Favera R. (1986). Chromosomal breakpoints and structural alterations of the c-myc locus differ in endemic and sporadic forms of Burkitt lymphoma. Proc. Natl. Acad. Sci. USA.

[278] Vaux D.L., Cory S., Adams J.M. (1988). Bcl-2 gene promotes haemopoietic cell survival and cooperates with c-myc to immortalize pre-B cells. Nature.

[279] Shimizu N., Tanabe-Tochikura A., Kuroiwa Y., Takada K. (1994). Isolation of Epstein-Barr virus (EBV)-negative cell clones from the EBV-positive Burkitt’s lymphoma (BL) line Akata: Malignant phenotypes of BL cells are dependent on EBV. J. Virol..

[280] Komano J., Sugiura M., Takada K. (1998). Epstein-Barr virus contributes to the malignant phenotype and to apoptosis resistance in Burkitt’s lymphoma cell line Akata. J. Virol..

[281] Chodosh J., Holder V.P., Gan Y.J., Belgaumi A., Sample J., Sixbey J.W. (1998). Eradication of latent Epstein-Barr virus by hydroxyurea alters the growth-transformed cell phenotype. J. Infect. Dis..

[282] Kirchmaier A.L., Sugden B. (1997). Dominant-negative inhibitors of EBNA-1 of Epstein-Barr virus. J. Virol..

[283] Vereide D., Sugden B. (2009). Proof for EBV’s sustaining role in Burkitt’s lymphomas. Semin. Cancer Biol..

[284] Vereide D.T., Sugden B. (2011). Lymphomas differ in their dependence on Epstein-Barr virus. Blood.

[285] Fitzsimmons L., Boyce A.J., Wei W., Chang C., Croom-Carter D., Tierney R.J., Herold M.J., Bell A.I., Strasser A., Kelly G.L. (2017). Coordinated repression of BIM and PUMA by Epstein-Barr virus latent genes maintains the survival of Burkitt lymphoma cells. Cell Death Differ..

[286] Ruf I.K., Rhyne P.W., Yang H., Borza C.M., Hutt-Fletcher L.M., Cleveland J.L., Sample J.T. (1999). Epstein-barr virus regulates c-MYC, apoptosis, and tumorigenicity in Burkitt lymphoma. Mol. Cell. Biol..

[287] Komano J., Maruo S., Kurozumi K., Oda T., Takada K. (1999). Oncogenic role of Epstein-Barr virus-encoded RNAs in Burkitt’s lymphoma cell line Akata. J. Virol..

[288] Ruf I.K., Rhyne P.W., Yang C., Cleveland J.L., Sample J.T. (2000). Epstein-Barr virus small RNAs potentiate tumorigenicity of Burkitt lymphoma cells independently of an effect on apoptosis. J. Virol..

[289] Fukuda M., Longnecker R. (2004). Latent membrane protein 2A inhibits transforming growth factor-beta 1-induced apoptosis through the phosphatidylinositol 3-kinase/Akt pathway. J. Virol..

[290] Bieging K.T., Amick A.C., Longnecker R. (2009). Epstein-Barr virus LMP2A bypasses p53 inactivation in a MYC model of lymphomagenesis. Proc. Natl. Acad. Sci. USA.

[291] Bieging K.T., Swanson-Mungerson M., Amick A.C., Longnecker R. (2010). Epstein-Barr virus in Burkitt’s lymphoma: A role for latent membrane protein 2A. Cell Cycle.

[292] Bell A.I., Groves K., Kelly G.L., Croom-Carter D., Hui E., Chan A.T., Rickinson A.B. (2006). Analysis of Epstein-Barr virus latent gene expression in endemic Burkitt’s lymphoma and nasopharyngeal carcinoma tumour cells by using quantitative real-time PCR assays. J. Gen. Virol..

[293] Tao Q., Robertson K.D., Manns A., Hildesheim A., Ambinder R.F. (1998). Epstein-Barr virus (EBV) in endemic Burkitt’s lymphoma: Molecular analysis of primary tumor tissue. Blood.

[294] Xue S.A., Labrecque L.G., Lu Q.L., Ong S.K., Lampert I.A., Kazembe P., Molyneux E., Broadhead R.L., Borgstein E., Griffin B.E. (2002). Promiscuous expression of Epstein-Barr virus genes in Burkitt’s lymphoma from the central African country Malawi. Int. J. Cancer.

[295] Choy E.Y., Siu K.L., Kok K.H., Lung R.W., Tsang C.M., To K.F., Kwong D.L., Tsao S.W., Jin D.Y. (2008). An Epstein-Barr virus-encoded microRNA targets PUMA to promote host cell survival. J. Exp. Med..

[296] Marquitz A.R., Mathur A., Nam C.S., Raab-Traub N. (2011). The Epstein-Barr Virus BART microRNAs target the pro-apoptotic protein Bim. Virology.

[297] Pimienta G., Fok V., Haslip M., Nagy M., Takyar S., Steitz J.A. (2015). Proteomics and Transcriptomics of BJAB Cells Expressing the Epstein-Barr Virus Noncoding RNAs EBER1 and EBER2. PLoS ONE.

[298] Coloff J.L., Mason E.F., Altman B.J., Gerriets V.A., Liu T., Nichols A.N., Zhao Y., Wofford J.A., Jacobs S.R., Ilkayeva O. (2011). Akt requires glucose metabolism to suppress puma expression and prevent apoptosis of leukemic T cells. J. Biol. Chem..

[299] Wu B., Guo B., Kang J., Deng X., Fan Y., Zhang X., Ai K. (2016). Downregulation of Smurf2 ubiquitin ligase in pancreatic cancer cells reversed TGF-beta-induced tumor formation. Tumour Biol..

[300] Spender L.C., Carter M.J., O’Brien D.I., Clark L.J., Yu J., Michalak E.M., Happo L., Cragg M.S., Inman G.J. (2013). Transforming Growth Factor-β directly induces PUMA during the rapid induction of apoptosis in Myc-driven B-cell lymphomas. J. Biol. Chem..

[301] Flavell J.R., Baumforth K.R., Wood V.H., Davies G.L., Wei W., Reynolds G.M., Morgan S., Boyce A., Kelly G.L., Young L.S. (2008). Down-regulation of the TGF-beta target gene, PTPRK, by the Epstein-Barr virus encoded EBNA1 contributes to the growth and survival of Hodgkin lymphoma cells. Blood.

[302] Kelly G.L., Milner A.E., Baldwin G.S., Bell A.I., Rickinson A.B. (2006). Three restricted forms of Epstein-Barr virus latency counteracting apoptosis in c-myc-expressing Burkitt lymphoma cells. Proc. Natl. Acad. Sci. USA.

[303] Kelly G., Bell A., Rickinson A. (2002). Epstein-Barr virus-associated Burkitt lymphomagenesis selects for downregulation of the nuclear antigen EBNA2. Nat. Med..

[304] Obexer P., Hagenbuchner J., Rupp M., Salvador C., Holzner M., Deutsch M., Porto V., Kofler R., Unterkircher T., Ausserlechner M.J. (2009). p16INK4A sensitizes human leukemia cells to FAS- and glucocorticoid-induced apoptosis via induction of BBC3/Puma and repression of MCL1 and BCL2. J. Biol. Chem..

[305] Garibal J., Hollville E., Bell A.I., Kelly G.L., Renouf B., Kawaguchi Y., Rickinson A.B., Wiels J. (2007). Truncated form of the Epstein-Barr virus protein EBNA-LP protects against caspase-dependent apoptosis by inhibiting protein phosphatase 2A. J. Virol..

[306] Cancer Genome Atlas Research Network (2014). Comprehensive molecular characterization of gastric adenocarcinoma. Nature.

[307] Ribeiro J., Oliveira C., Malta M., Sousa H. (2017). Epstein-Barr virus gene expression and latency pattern in gastric carcinomas: A systematic review. Future Oncol..

[308] Hu L., Lin Z., Wu Y., Dong J., Zhao B., Cheng Y., Huang P., Xu L., Xia T., Xiong D. (2016). Comprehensive profiling of EBV gene expression in nasopharyngeal carcinoma through paired-end transcriptome sequencing. Front. Med..

[309] Zhu S., Sun P., Zhang Y., Yan L., Luo B. (2013). Expression of c-myc and PCNA in Epstein-Barr virus-associated gastric carcinoma. Exp. Ther. Med..

[310] Zur Hausen A., Brink A.A., Craanen M.E., Middeldorp J.M., Meijer C.J., van den Brule A.J. (2000). Unique transcription pattern of Epstein-Barr virus (EBV) in EBV-carrying gastric adenocarcinomas: Expression of the transforming BARF1 gene. Cancer Res..

[311] Cabras G., Decaussin G., Zeng Y., Djennaoui D., Melouli H., Broully P., Bouguermouh A.M., Ooka T. (2005). Epstein-Barr virus encoded BALF1 gene is transcribed in Burkitt’s lymphoma cell lines and in nasopharyngeal carcinoma’s biopsies. J. Clin. Virol..

[312] Strockbine L.D., Cohen J.I., Farrah T., Lyman S.D., Wagener F., DuBose R.F., Armitage R.J., Spriggs M.K. (1998). The Epstein-Barr virus BARF1 gene encodes a novel, soluble colony-stimulating factor-1 receptor. J. Virol..

[313] Hoebe E.K., Le Large T.Y., Tarbouriech N., Oosterhoff D., De Gruijl T.D., Middeldorp J.M., Greijer A.E. (2012). Epstein-Barr virus-encoded BARF1 protein is a decoy receptor for macrophage colony stimulating factor and interferes with macrophage differentiation and activation. Viral. Immunol..

[314] Dolcetti R. (2015). Cross-talk between Epstein-Barr virus and microenvironment in the pathogenesis of lymphomas. Semin. Cancer Biol..

[315] Concha M., Wang X., Cao S., Baddoo M., Fewell C., Lin Z., Hulme W., Hedges D., McBride J., Flemington E.K. (2012). Identification of new viral genes and transcript isoforms during Epstein-Barr virus reactivation using RNA-Seq. J. Virol..

[316] Fox C.P., Haigh T.A., Taylor G.S., Long H.M., Lee S.P., Shannon-Lowe C., O’Connor S., Bollard C.M., Iqbal J., Chan W.C. (2010). A novel latent membrane 2 transcript expressed in Epstein-Barr virus-positive NK- and T-cell lymphoproliferative disease encodes a target for cellular immunotherapy. Blood.

[317] Yuen K.S., Chan C.P., Kok K.H., Jin D.Y. (2017). Mutagenesis and Genome Engineering of Epstein-Barr Virus in Cultured Human Cells by CRISPR/Cas9. Methods Mol. Biol..

[318] Pujals A., Favre L., Pioche-Durieu C., Robert A., Meurice G., Le Gentil M., Chelouah S., Martin-Garcia N., Le Cam E., Guettier C. (2015). Constitutive autophagy contributes to resistance to TP53-mediated apoptosis in Epstein-Barr virus-positive latency III B-cell lymphoproliferations. Autophagy.

[319] Lee D.Y., Sugden B. (2008). The latent membrane protein 1 oncogene modifies B-cell physiology by regulating autophagy. Oncogene.

[320] Brune W., Andoniou C.E. (2017). Die Another Day: Inhibition of Cell Death Pathways by Cytomegalovirus. Viruses.

[321] Veyer D.L., Carrara G., Maluquer de Motes C., Smith G.L. (2017). Vaccinia virus evasion of regulated cell death. Immunol. Lett..

[322] Kofahi H.M., Taylor N.G., Hirasawa K., Grant M.D., Russell R.S. (2016). Hepatitis C Virus Infection of Cultured Human Hepatoma Cells Causes Apoptosis and Pyroptosis in Both Infected and Bystander Cells. Sci. Rep..

